# Large-scale metagenomic surveillance study expands the known diversity of RNA viruses in mosquito populations from the Amazon Basin

**DOI:** 10.7717/peerj.20880

**Published:** 2026-03-11

**Authors:** Eddie Fuques, Aimee L. Massey, Faris Qureshi, João Vitor Campos-Silva, David J. Ferreira da Silva, Carlos A. Peres, Taal Levi, Rebecca L. Vega Thurber

**Affiliations:** 1 Microbiology, Oregon State University, Corvallis, OR, United States of America; 2 Ecology, Evolution, and Marine Biology, University of California, Santa Barbara, Santa Barbara, CA, United States of America; 3 Fisheries, Wildlife, and Conservation Sciences, Oregon State University, Corvallis, OR, United States of America; 4 Instituto Juruá, Carauari, Amazonas, Brazil; 5 Instituto de Ciências da Saúde, Universidade Federal de Mato Grosso, Sinop, Mato Grosso, Brazil; 6 School of Environmental Sciences, University of East Anglia, Norwich, Norwich, United Kingdom

**Keywords:** RNA viruses, Mosquito virome, Amazon Basin, Viral ecology, Insect-specific viruses

## Abstract

The Amazon Basin is one of the most biologically diverse regions on Earth, yet its viral diversity remains poorly characterized. Mosquitoes are important vectors and reservoirs of RNA viruses, but little is known about the composition and structure of their viromes in remote areas of the Amazon. In this study, we performed a large-scale metagenomics survey of RNA viruses associated with mosquito populations collected from the Jurua River region in the Western Amazon Basin of Brazil. We analyzed 211 pooled samples of adult female mosquitoes collected across thirty-seven sites, representing one of the most comprehensive mosquito virome studies conducted in this region to date. Utilizing high-throughput sequencing and *de novo* assembly, we identified over 500 viral sequences from 18 families, including 21 complete or nearly complete genomes. Our analysis revealed 18 putative novel viral species spanning diverse families and strains of nine previously described viruses. Phylogenetic analyses also revealed undocumented diversity within several virus families, including *Iflaviridae, Mesoniviridae, Phasmaviridae, Phenuiviridae, Togaviridae,* and *Totiviridae,* encompassing both novel species and previously known viruses detected for the first time in this region. Our findings highlight the immense, yet largely unexplored, diversity of RNA viruses circulating in mosquito populations in this ecologically rich but understudied region and provide critical insights into the evolutionary dynamics of mosquito-associated viruses. By leveraging high-throughput sequencing to uncover novel viral strains, this research demonstrates the value of metagenomic approaches in expanding the known diversity, distribution, and evolutionary relationships of RNA viruses, contributing to a broader understanding of virus-mosquito interactions and genome evolution.

## Introduction

Arthropods play a crucial role in the transmission of pathogens, posing significant risks to both human and animal health. Among these, mosquitoes are particularly concerning as vectors for numerous pathogens and hosts of a variety of underexplored and potentially emerging viral diseases. These include arthropod-borne viruses (arboviruses), which cycle between mosquitoes and vertebrate hosts, as well as insect-specific viruses (ISVs), which are restricted to insect hosts (Bolling et al., 2015; Weaver & Reisen, 2010). RNA viruses, known for their high mutation rates and genetic diversity, are particularly prevalent among mosquito-borne viruses. Notable examples include dengue (DEN), chikungunya (CHIK), yellow fever (YF), and West Nile (WN) viruses, making arboviruses a primary focus of viral monitoring and human health research.

The high abundance and diversity of RNA viruses reported in mosquitoes support the idea that arthropods may have played a significant role in viral evolution, potentially serving as reservoirs where insect-specific viruses could evolve into dual-host viruses capable of infecting both insects and vertebrates (Öhlund, Lundén & Blomström, 2019). The ecological complexity and the interaction between these vectors and their environment create opportunities for viral diversity to emerge and persist, with direct implications for public health and conservation (Liu et al., 2023). Consequently, understanding the diversity and dynamics of RNA viruses in mosquito populations is essential for discerning viral evolution and assessing the potential for spillover events, aiding surveillance efforts, and facilitating the early detection and mitigation of emerging infectious diseases.

In recent years, the development of advanced sequencing technologies, such as high-throughput sequencing (HTS), has transformed our ability to analyze viral communities (viromes) across diverse hosts and environments (Dominguez-Huerta et al., 2023). These methodological advancements have proven crucial for examining mosquito-associated viruses and their ecological contexts, particularly in remote and lesser-studied geographical areas (Liu et al., 2023; Moonen et al., 2023). Surveillance efforts have continually expanded our understanding of viral diversity and evolution, revealing new host associations, novel viral lineages, and even new families (Bolling et al., 2015; Hermanns et al., 2023; Hollingsworth et al., 2023; Sadeghi et al., 2018). These discoveries illustrate that the tremendous diversity of viral communities in mosquitoes is just being uncovered and highlight the significance of continued research to explore this complexity and its potential impact on animal and human disease.

Biodiverse and ecologically complex regions are essential for understanding the full scope of virus-host interactions and the factors that shape viral communities. Mosquitoes in these areas interact with a diverse range of hosts and environmental conditions, facilitating viral evolution and potential cross-species transmission. Studying these ecosystems not only expands our knowledge of viral evolution and host associations but also helps establish a more complete picture of how viruses are distributed across a range of different habitats. Given the limited surveillance efforts in many of these remote regions, targeted investigations are necessary to uncover the extent of viral diversity and its broader ecological significance.

The Amazon Basin constitutes one of the most biologically rich and complex regions on Earth, harboring a vast array of ecosystems and species, including over 100 mosquito species (De Aquino et al., 2022; Garda, Da Silva & Baião, 2010; Hutchings et al., 2018; Mittermeier et al., 2003). This immense diversity presents a unique opportunity for studying these interactions as the human footprint continues to expand into these largely uninhabited regions, potentially leading to spillover events when communities increasingly interact with wildlife. In this study, we conducted a broad and deep survey of RNA viruses from mosquito populations from the Jurua River region in the Western Amazon Basin. Using HTS and *de novo* assembly, we analyzed 211 mosquito pools collected across a river transect of approximately 150 Kilometers in the Amazon Basin, encompassing a variety of habitats. This effort resulted in the classification of 51 viral Metagenomic Assembled Genomes (vMAGs) into 18 tentative novel viral species from a diverse range of families and 33 viral sequences as strains of nine previously described viruses. Our analysis focused on characterizing the diversity and abundance of these viruses, circulating in this ecologically rich yet understudied area. This research provides new insights into the viral diversity present in Amazonian mosquitoes, offering a broader perspective on the range of RNA viruses associated with mosquito populations in this region. By characterizing viral genomes from pooled mosquito samples, this study expands the known diversity of RNA viruses and highlights the potential ecological roles of mosquitoes in viral maintenance and transmission.

## Materials and Methods

### Samples collection

**Figure 1:**
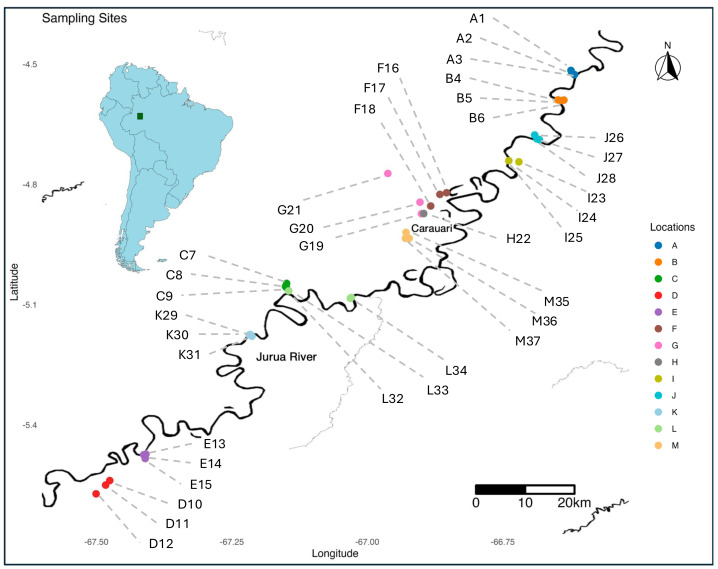
Map of the region showing the sampling sites along the Jurua River. Locations are marked with different colors, corresponding to the legend on the right. The insert in the top left provides geographic context, with the study area highlighted by a green square in a zoomed-out map of South America.

Mosquito sampling was conducted during two field seasons across 13 locations along the Jurua River region in the Western Amazon (Fig. 1). These locations were designated A through M, with multiple sampling sites identified within each location. Locations were sampled in alphabetical order; A to H were sampled from mid-September through October in 2021, while locations I to M were sampled from mid-September through mid-October in 2022. Each site was assigned a unique alphanumeric code to delineate specific areas (*e.g.*, A1, A2, A3, B4, B5, B6, *etc*.). Additionally, multiple mosquito pools were collected at each sampling site, serving as replicates for the study (*e.g* A1_13, A1_28, B4_20, *etc*.).

To maximize mosquito capture, 12 CDC UV light traps were deployed per grid at each site for a total of 36 traps per location. Mosquitoes were trapped over two consecutive trap nights (∼48 h) and collected every 24 h, resulting in two discrete collections per trap. Following collection, mosquitoes were sorted at the trap and trap night level to isolate females, and then pooled at 50 individual females per sample and stored in RNA/DNA Shield (Zymo Research, Irvine, CA, USA) to preserve nucleic acids for sequencing. In total, we processed 211 mosquito pools from all 37 different sampling sites for RNA sequencing (Fig. 1, Table S1).

Field sample collections were conducted under permits issued by the Instituto Chico Mendes de Conservacão da Biodiversidade (ICMBio) in 2021 and by the Secretaria de Estado do Meio Ambiente do Amazonas (project authorization number 034/2022) in 2022.

### Samples processing

Total nucleic acids were extracted from the mosquito pools using the Quick-DNA-RNA Pathogen Miniprep Kit (Zymo Research, Irvine, CA, USA). rRNA depletion and RNA sequencing library preparation were performed using the Illumina Stranded Total RNA Prep with Ribo-Zero Plus kit (Illumina, San Diego, CA, USA). The libraries were sequenced using two full flow cells of the Illumina NovaSeq 6000 platform at the University of Oregon’s Genomics and Cell Characterization Core Facility (GC3F). The sequencing was performed on S4 flow cells in paired-end format with a read length of 2 × 150 bp.

### Data analysis

Raw reads quality was assessed with fastQC v0.11.9 (Andrews, 2010) and multiQC v1.14 (Ewels et al., 2016). Trimming of the reads was performed using fastp v0.23.2 (S. Chen et al., 2018) with the following parameters: minimum average quality of 25, minimum base quality of 20, minimum read length of 50, minimum complexity of 30%, remove poly-A, maximum of 2 Ns, auto-detect and remove adapters. Trimmed paired-end reads were subjected to a normalization step using bbnorm tool from BBTools (Bushnell, 2023), in order to reduce the number of total reads before assembling (target = 40, min = 0; all other parameters default). *De novo* assembly was performed using rnaSPAdes v3.15.3 (Bushmanova et al., 2019) with k-mer values 77, 99, and 127, selected to balance sensitivity and accuracy. Obtained contigs were filtered by length (minimum length of 1,000 bp) and used to perform a BLASTX search using DIAMOND v2.1.9 (Buchfink, Reuter & Drost, 2021) in sensitive mode and the latest RefSeq viral protein database (as of June 2025). A minimum bitscore of 300 and a 70% identity were used as a threshold to classify contigs as viral according to the BLASTX outputs. Viral protein sequences were obtained using Prodigal v2.6.3 (Hyatt et al., 2010) in metagenomic mode (*-p meta*) using NCBI standard genetic code 1 (-g 1), with default settings otherwise. CheckV (Nayfach et al., 2021) was run on viral contigs as an additional verification step to screen for potential contamination and to summarize hallmark-gene evidence alongside our Prodigal annotations. For single-contig genomes, CheckV completeness estimates were used as indicators and are provided for context (Table S2). Raw reads were mapped back to viral contigs and quantified using Kallisto v0.48.00 (Bray et al., 2016), which reports abundance as transcripts per million (TPM). TPM normalizes estimated counts by contig length and by overall sequencing depth, providing normalized measures of viral abundance.

For each putative viral contig, the family of the top hit was recorded as a candidate classification. For family-level assignment, we then built maximum-likelihood phylogenies of the RNA-dependent RNA polymerase (RdRp) or the appropriate ORF (*e.g.*, polyprotein ORF for *Iflaviridae*) that included representative members of the family together with outgroup taxa from related families. Sequences that clustered with strong support within the corresponding family clade were assigned to that family. Within these family-level assignments, tentative new viruses were designated using 95% amino acid identity in the analysed protein sequence (RdRp, or the polyprotein for *Iflaviridae*) as a primary criterion, supported by phylogenetic analyses to confirm their evolutionary relationship. This threshold is directly in line with International Committee on Taxonomy of Viruses (ICTV) species demarcation criteria for *Phasmaviridae* and *Phenuiviridae* (L/RdRp 95% amino acid identity between species) and is more conservative than the 90% capsid protein identity used between species in *Iflaviridae*. For the remaining families in which we identified putatively novel viruses (*e.g.*, *Togaviridae*, *Mesoniviridae*, *Totiviridae*), for which ICTV does not define a single RdRp/polyprotein cutoff, we used this 95% identity threshold as a conservative indicator of potential novelty, interpreted together with phylogenetic context. Additional factors, including associated Genbank metadata such as sampling location, collection date, and the host organism, were also considered to assess their novelty.

For contigs whose only viral matches were to poorly characterized hypothetical proteins and that lacked supported genome segments or hallmark genes, we performed additional BLAST searches against the NCBI nr database. Contigs whose best hits were to non-viral proteins (*e.g.*, insect genes) were considered host-derived and removed from the viral dataset.

Multiple sequence alignments (MSA) were performed with mafft v7.520 (Katoh & Standley, 2013) with the–auto parameter using protein sequences from this study and reference sequences retrieved from the GenBank database. When multiple sequences were highly similar, CD-HIT v4.8.1 (Fu et al., 2012) was applied with a 98% identity threshold to retain only representative sequences. MSAs were trimmed using ClipKit v2.3.0 (Steenwyk et al., 2020) in the *kpi-smart-gap* mode to retain only parsimony-informative sites. Substitution model selection and phylogenetic analysis were performed using IQ-TREE 2 (Minh et al., 2020) applying 1,000 bootstrap replicates (*-b 1000*) and 1,000 replicates for the approximate likelihood ratio test (*-alrt 1000*). Phylogenetic trees were visualized and edited using iTOL v6 (Letunic & Bork, 2024).

## Results

### Mosquito capture, sorting, and pooling reveals a diverse population of RNA viral Metagenomic Assembled Genomes (vMAGs)

A total of 10,550 mosquitoes were collected from 13 locations across the Jurua river region in the Western Amazon, from which 211 pools of fifty putative female mosquitoes were created, resulting in 211 RNAseq libraries (one of the largest datasets of its kind, NCBI BioProject PRJNA1223432). High-throughput sequencing generated approximately 4.3 TB of sequencing data, covering a ∼150-kilometer transect of the Jurua River (Fig. 1). This dataset provided a comprehensive foundation for identifying and characterizing the RNA viral diversity associated with mosquitoes in this ecologically complex and understudied region.

Mosquito RNAseq libraries had an average of 26,597,204 high-quality paired reads per library (Table S3). Following *de novo* assembly, 209 libraries contained contigs longer than 1 Kb, with 138 of these containing at least one >1 Kb contig classified as viral based on conservative DIAMOND and CheckV results, as described in methods. While all the locations (A to M) covered in this study remained represented in the final 138 libraries used for viral population analyses, the number of total sampling sites was reduced from 37 to 35, as libraries from two sampling sites (K_29 and L_33) did not contain sufficiently high-quality viral contigs.

**Figure 2:**
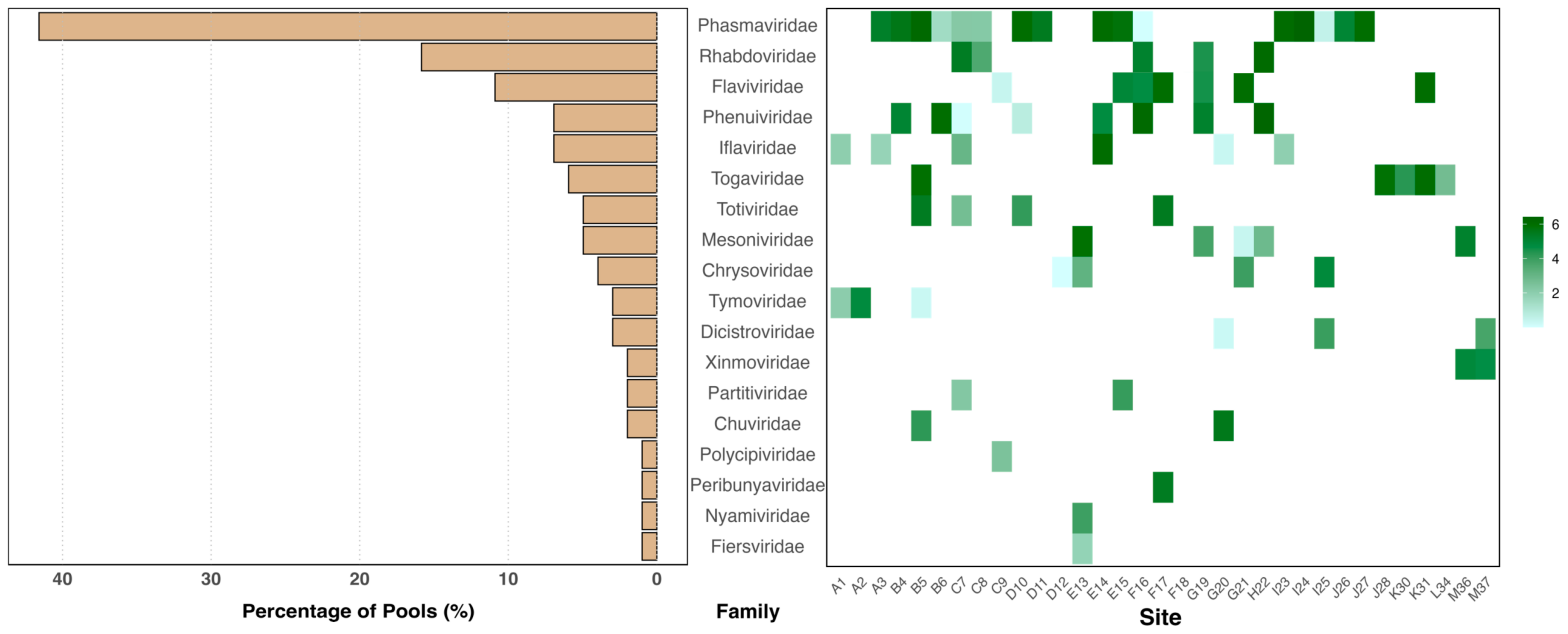
Overall virus diversity and abundance . The bar chart on the left shows the prevalence of viral families identified in this study across the different pools (libraries). The heatmap on the right illustrates their respective abundances (represented as Log(TPM+1)) across the sampling sites.

BLASTX results identified 650 viral contigs longer than 1 Kb (based on parameters described in the methods), representing 18 different viral RNA families (Fig. 2). The most prevalent viral family was *Phasmaviridae*, identified in over 40% of the pools. *Rhabdoviridae* and *Flaviviridae* were found in 10–20% of the pools, while families *Phenuiviridae*, *Iflaviridae*, and *Togaviridae* were detected in 5–10%. CheckV results indicated that 51 viral Metagenomic Assembled Genomes (vMAGs) had a completeness level greater than 95%, with thirty-four showing 100% completeness.

Within the identified viral families, 25 contigs were classified as belonging to the family *Retroviridae*. However, due to the challenges in distinguishing between endogenous and exogenous retroviruses in this type of sequencing data, these sequences were excluded from further analysis and are not included in the results presented here.

### Genetic characterization of viral sequences reveals unknown diversity of viral families in the Amazonas Basin

Protein sequences derived from the vMAGs were used for phylogenetic analyses together with representative reference sequences to compare our viruses with previously reported strains and to resolve within-family diversity (see Methods). Based on the criteria described above, a total of 82 viral strains were classified as either novel viruses or strains from previously reported viruses (Table 1). This section presents the results for viral families identified, focusing on sequences that were sufficiently long and overlapping to allow for MSA and phylogenetic analyses, enabling their assignment to a viral taxon.

**Table 1:** Viral strains obtained in this study that were classified at a species level. Corresponding families, pools from which they were obtained, strain names, and GenBank accession numbers.

***Bunyavirales***				
**Family**	**Virus name**	**Pool**	**Strain**	**GenBank accession**
*Phasmaviridae*	Jurua Jon-like virus^*^	A3_2	A3_2/780/BR2021	PV269761
	Anopheles triannulatus Orthophasmavirus	B6_22	B6_22/775/BR2021	PV269760
	Jurua Orthophasmavirus^*^	J27_25	J27_25/5/BR2022	PV269766
	Coredo virus	E15_1	E15_1/19/BR2021	PV269768
		B5_77	B5_77/9/BR2021	PV269767
	Coredo-like virus 1^*^	B5_87	B5_87/7/BR2021	PV274264
		B5_84	B5_84/7/BR2021	PV274263
		D10_17	D10_17/30/BR2021	PV274265
		D11_14	D11_14/100/BR2021	PV274268
		D11_2	D11_2/141/BR2021	PV274266
		D11_6	D11_6/167/BR2021	PV274267
		I24_22	I24_22/11/BR2022	PV274269
	Coredo-like virus 2^*^	C7_12	C7_12/19/BR2021	PV274270
		C8_15	C8_15/82/BR2021	PV274271
		D10_8	D10_8/18/BR2021	PV274273
		D10_16	D10_16/3/BR2021	PV274272
		E14_14	E14_14/35/BR2021	PV274274
		F16_7	F16_7/34/BR2021	PV274275
	Coredo-like virus 3^*^	B4_59	B4_59/8/BR2021	PV287660
		B4_96	B4_96/22/BR2021	PV287663
		B5_100	B5_100/3/BR2021	PV287664
		B5_2	B5_2/984/BR2021	PV287665
		I24_20	I24_20/37/BR2022	PV287668
		I24_2	I24_2/44/BR2022	PV287669
		J26_21	J26_21/216/BR2022	PV287673
		J26_9	J26_9/180/BR2022	PV287674
		J27_24	J27_24/85/BR2022	PV287675
		A3_18	A3_18/51/BR2021	PV287659
		I23_2	I23_2/148/BR2022	PV287666
		I24_5	I24_5/15/BR2022	PV287670
		J26_19	J26_19/389/BR2022	PV287672
		B4_60	B4_60/13/BR2021	PV287661
		I25_31	I25_31/6/BR2022	PV287671
		B4_82	B4_82/35/BR2021	PV287662
		I23_87	I23_87/2/BR2022	PV287667
*Phenuiviridae*	Jurua Narangue-like virus^*^	B4_1	B4_1/182/BR2021	PV287676
	Jurua Mobuvirus^*^	D10_2	D10_2/25/BR2021	PV287678
		C7_2	C7_2/2/BR2021	PV287677
	Jurua Cumuto-like virus^*^	B6_22	B6_22/2/BR2021^**^	PV335187
	Jurua Goukovirus^*^	E14_28	E14_28/17/BR2021	PV335190
*Peribunyaviridae*	Alajuela virus	F17_5	F17_5/2/BR2021^**^	PV335191
***Mononegavirales***				
**Family**	**Virus name**	**Pool**	**Strain**	**GenBank accession**
*Rhabdoviridae*	Merida virus	C8_31	C8_31/BR2021^**^	PV335215
		C7_13	C7_13/BR2021^**^	PV357251
		F16_1	F16_1/BR2021^**^	PV357252
		G19_1	G19_1/BR2021^**^	PV335218
		F16_2	F16_2/BR2021^**^	PV335216
		F16_9	F16_9/BR2021^**^	PV335217
		G19_4	G19_4/BR2021^**^	PV335219
		G19_2	G19_2/BR2021	PV357253
		H22_1	H22_1/BR2021^**^	PV335220
		H22_2	H22_2/BR2021	PV357254
		H22_3	H22_3/BR2021	PV335222
		H22_5	H22_5/BR2021	PV357255
		H22_8	H22_8/BR2021	PV335223
		H22_9	H22_9/BR2021	PV357256
		H22_10	H22_10/BR2021	PV357257
		H22_12	H22_12/BR2021^**^	PV335221
*Xinmoviridae*	Anopheles darlingui virus	M36_2	M36_2/2/BR2022^**^	PV335194
		M37_4	M37_4/1/BR2022^**^	PV335195
***Picornavirales***				
**Family**	**Virus name**	**Pool**	**Strain**	**GenBank accession**
*Iflaviridae*	Opsiphanes invirae iflavirus 1	E14_41	E14_41/1/BR2021^**^	PV335196
	Jurua Iflavirus 1^*^	G20_6	G20_6/1/BR2021^**^	PV335197
	Jurua Iflavirus 2^*^	I23_2	I23_2/1/BR2022	PV335198
	Jurua Iflavirus 3^*^	C7_10	C7_10/152/BR2021	PV335199
	Jurua Iflavirus 4^*^	E14_28	E14_28/46/BR2021	PV335214
		A1_28	A1_28/522/BR2021	PV335213
***Amarillovirales***				
**Family**	**Virus name**	**Pool**	**Strain**	**GenBank accession**
*Flaviviridae*	Culex flavivirus	G19_2	G19_2/50/BR2021	PV335202
		C9_17	C9_17/18/BR2021	PV335200
		F16_9	F16_9/7/BR2021	PV335201
		G21_2	G21_2/91/BR2021	PV335203
***Nidovirales***				
**Family**	**Virus name**	**Pool**	**Strain**	**GenBank accession**
*Mesoniviridae*	Alphamesonivirus 1	H22_5	H22_5/2/BR2021	PV357258
		G19_1	G19_1/1/BR2021^**^	PV335224
	Carauari Mesonivirus^*^	M36_2	M36_2/1/BR2021^**^	PV335225
	Jurua Mesonivirus^*^	G21_1	G21_1/1/BR2021^**^	PV335226
***Togaviridae***				
**Family**	**Virus name**	**Pool**	**Strain**	**GenBank accession**
*Togaviridae*	Caaingua virus	K30_25	K30_25/2/BR2022	PV335204
		K31_1	K31_1/1/BR2022^**^	PV335205
		L34_14	L34_14/1/BR2022^**^	PV335207
		K31_30	K31_30/1/BR2022	PV335206
	Jurua Alphavirus^*^	B5_100	B5_100/1/BR2021^**^	PV335208
***Ghabrivirales***				
**Family**	**Virus name**	**Pool**	**Strain**	**GenBank accession**
*Totiviridae*	Jurua Totivirus 1^*^	B5_76	B5_76/37/BR2021	PV335209
	Jurua Totivirus 2^*^	F17_2	F17_2/263/BR2021	PV335212
		D10_1	D10_1/127/BR2021	PV335210
		D10_2	D10_2/1706/BR2021	PV335211

**Notes.**

Tentative novel viruses are marked with an asterisk (*).

Strains where complete genome/segments were obtained are marked with a double asterisk (**).

#### Phasmaviridae

We identified ninety-eight contigs associated with the *Phasmaviridae* family from forty-two pools across sixteen different sampling sites. Members of this family are characterized by a segmented genome with three negative-sense RNA segments (L, M, and S). BLASTX analysis revealed best hits to Jonchet virus (one sequence), Anopheles orthophasmavirus (one sequence), Wuhan mosquito virus 1 (one sequence), and Coredo virus (ninety-five sequences). Among these, seventy-three sequences corresponded to the L segment, from which thirty-nine RdRp sequences were used to construct an MSA alongside representative sequences from all known *Phasmaviridae* genera.

**Figure 3:**
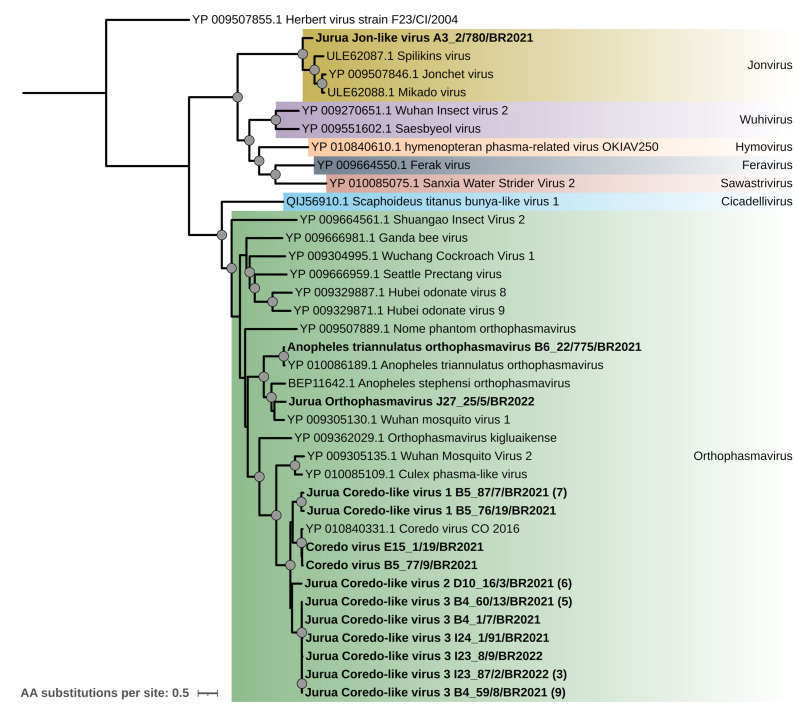
Maximum likelihood phylogenetic tree based on the L segment (2,066 aa sites) of the *Phasmaviridae* family. Sequences obtained in this study are shown in bold. Circles on nodes indicate bootstrap values ≥85, and colors represent the different genera within this family. Numbers within parentheses indicate the number of sequences represented by a single tip after clustering analysis using CD-HIT with a 98% aa identity threshold.

Phylogenetic analysis showed that the sequence obtained from pool A3_2 (strain A3_2/780/BR2021) clustered with other strains from the genus *Jonvirus* (Spilikin, Jonchet, and Mikado virus), forming a monophyletic clade with high statistical support (Fig. 3). The amino acid sequence identity between this strain and previously reported *Jonvirus* was 71.2%, 71.5%, and 72.0% with Mikado, Spilikins, and Jonchet viruses, respectively. We tentatively named this virus Jurua Jon-like virus.

The RdRp sequence obtained from pool B6_22 (strain B6_22/775/BR2021) shared 97% amino acid identity with Anopheles triannulatus orthophasmavirus, indicating that it corresponds to a variant of this virus. The sequence obtained from pool J27_25 (strain J27_25/5/BR2022) showed 76% identity with Wuhan mosquito virus 1. We named this tentative new virus Jurua orthophasmavirus. Phylogenetic analysis grouped these viral strains in a monophyletic clade within the genus *Orthophasmavirus* with high statistical support, alongside Anopheles stephensi orthophasmavirus (Fig. 3).

The thirty-six sequences closely related to Coredo virus were grouped into eleven clusters using CD-HIT analysis with a 98% amino acid identity threshold (Table S4). For better visualization, only representative sequences from each cluster were employed in the phylogenetic analysis. These sequences formed a major clade composed of Coredo and Coredo-related sequences. Using a 95% amino acid identity threshold, the sequences were further classified into four novel clades named Jurua Coredo-like virus 1–3 (Fig. 3; Table S4).

#### Phenuiviridae

**Figure 4:**
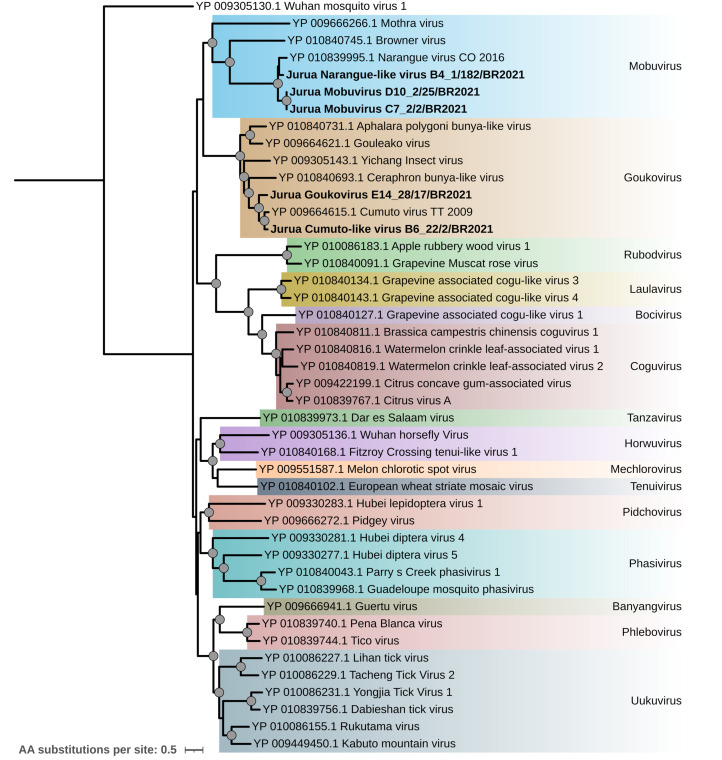
Maximum likelihood phylogenetic tree for the L segment (2,509 amino acid sites) from the *Phenuiviridae* family. Sequences obtained in this study are shown in bold. Circles on nodes indicate bootstrap values ≥85. Colors represent the different genera within this family.

Thirteen contigs from seven pools across six sampling sites were identified as belonging to the *Phenuiviridae* family. The most closely related known viral species were Narangue virus (five pools) and Cumuto virus (two pools). Five RdRp sequences were included in a phylogenetic analysis alongside representative sequences from the family (Fig. 4). The resulting phylogenetic tree revealed multiple clades, reflecting the family’s diversity and its classification into several genera.

Three viral sequences grouped within the genus *Mobuvirus* with high statistical support, representing two tentatively novel viruses closely related to Narangue virus: Jurua Narangue-like virus (strain B4_1/182/BR2021, 84% RdRp amino acid identity) and Jurua mobuvirus (strains C7_2/2/BR2021 and D10_2/25/BR2021, 73.3% and 71.4% RdRp amino acid identity, respectively).

Sequences from pools E14_28 and B6_22, closely related to Cumuto virus, clustered within the genus *Goukovirus* with high statistical support. These sequences correspond to two tentative new viruses: Jurua goukovirus and Jurua Cumuto-like virus, obtained from pools E14_28 and B6_22, respectively (Fig. 4). While both viruses had Cumuto virus as their best BLASTX hit, they showed distinct amino acid identities with their RdRp protein sequence: 74.7% for Jurua goukovirus and 83.5% for Jurua Cumuto-like virus.

#### Peribunyaviridae

All three genomic segments of a virus from the genus *Orthobunyavirus* were identified in pool F17_5. The segments were 6,934 bp (L segment), 5,044 bp (M segment), and 1,315 bp (S segment) in length. BLASTX analysis revealed amino acid identities of 98.1%, 83.7%, and 97.1% for the L, M, and S segments, respectively, compared to Alajuela virus. Based on the established 95% species threshold for the RdRp (L segment) and phylogenetic analysis, these results indicate that the L segment corresponds to a strain of previously described Alajuela virus reported in Argentina (Nunes et al., 2014).

#### Nairoviridae

Twelve contigs from twelve different mosquito pools were associated with the *Nairoviridae* family. For all these contigs, the best match was to a hypothetical protein encoded by the M segment of Blattodean nairo-related virus (also known as red goblin roach virus 1), with amino acid identities ranging from 72% to 75%. According to the ICTV, this hypothetical protein is characteristic of the genus *Ocetevirus*, the only genus within the *Nairoviridae* family where this protein has been reported. The identified contigs ranged in length from 1,035 bp to 1,775 bp. Notably, no contigs were associated with the L segment (encoding the RdRp protein) or the S segment (encoding the nucleocapsid protein) of the *Nairoviridae* genomes. Additionally, none of the contigs matched the Glycoprotein precursor, the other gene encoded by the M segment.

To evaluate alternative explanations, we further searched these twelve contigs against the NCBI nr database using BLASTN. In all cases, the best-scoring hit was a mosquito phosphatase gene (accession XM_038252539.1) showing nucleotide identity values greater than 93%. These results indicate that the contigs are most likely host-derived sequences rather than actual *Nairoviridae* segments. We therefore no longer treat them as evidence of *Nairoviridae* presence in our pools and have removed them from our viral catalog and all downstream analyses.

#### Rhabdoviridae

Forty-six contigs from sixteen pools were identified as belonging to the *Rhabdoviridae* family, with Merida virus (MERDV) as their best hit according to BLASTX results. Nine corresponded to full vMAGs (∼11 Kb), from which we could annotate the five genes that compose the genome and encode for the nucleoprotein, phosphoprotein, matrix protein, glycoprotein, and RdRp. Full-length RdRp protein sequences were employed for the genetic characterization of the strains. All sixteen RdRp sequences obtained from the complete and nearly complete genomes were highly similar, forming a single cluster with 98% amino acid identity threshold based on CD-HIT analysis (Fig. S1). Strain C7_13/BR2021 from pool C7_13 was employed as a representative sequence of this cluster for better visualization of the tree.

**Figure 5:**
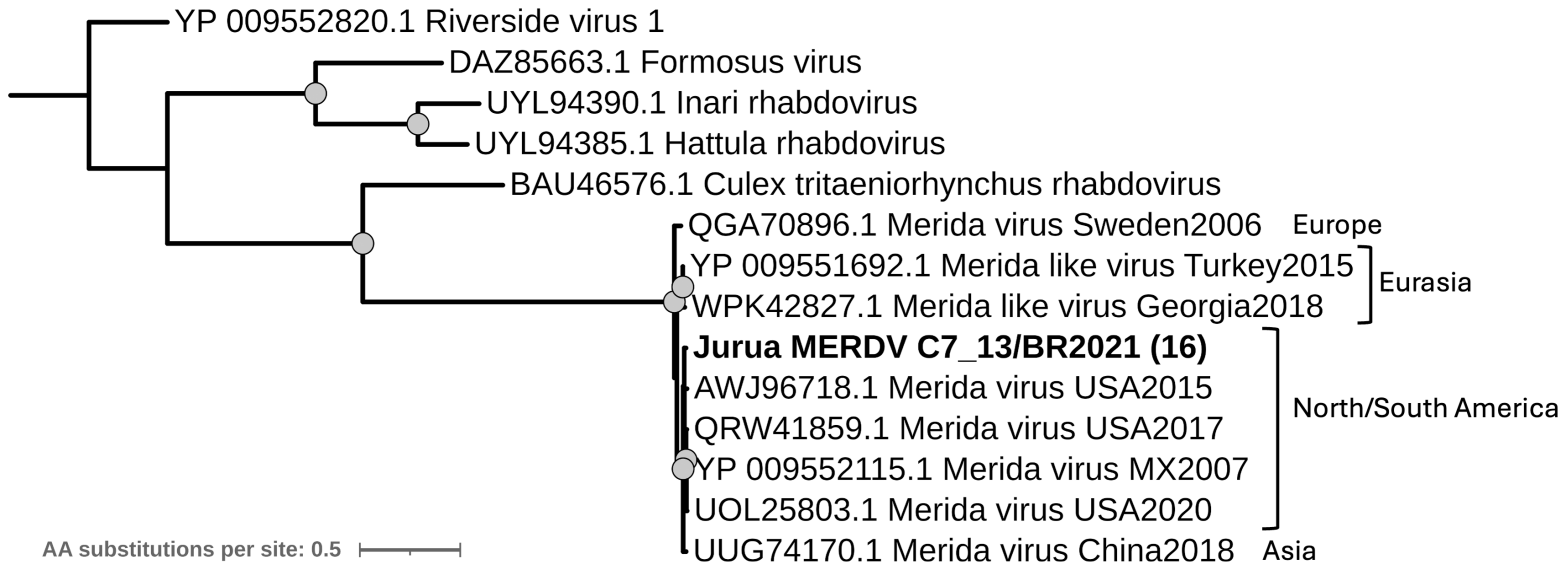
Maximum likelihood phylogenetic tree for the RdRp protein sequence (1,202 sites) from the *Merhavirus* genus. Sequences obtained in this study are shown in bold. Circles on nodes indicate bootstrap values ≥85. Numbers within parentheses indicate the number of sequences represented by a single tip after clustering analysis using CD-HIT with a 98% aa identity threshold. Geographic locations for the Merida virus strains are indicated.

Amino acid identities among the RdRp sequences showed that Merida virus sequences from this study present high amino acid identity (≥98%) with other Merida virus sequences from the Americas (USA and Mexico). In contrast, they exhibited slightly lower identity values (95–96%) with sequences from Europe (*e.g.*, Sweden, Georgia, and Turkey) and Asia (*e.g.*, China) (Fig. 5; Fig. S1).

#### Xinmoviridae

Two complete vMAGs were recovered from pools M36_2 and M37_4, both classified within the genus *Madalivirus*, which currently includes only two reported species: Anopheles darlingui virus and Anopheles marajoara virus. The recovered genomes correspond to strains of Anopheles darlingi virus, originally isolated in 2015 in Brazil (Scarpassa et al., 2019), with RdRp amino acid identities of 99.8% and 99.9% for pools M36_2 and M37_4, respectively. When compared to all available *Madalivirus* strains, the RdRp sequences of our genomes showed identity values greater than 98% with anopheles darlingi virus strains and greater than 70% with Anopheles marajoara virus strains (Fig. S2). Additionally, we were able to annotate all five previously reported Madalivirus genes in both genomes: nucleoprotein, transmembrane protein, glycoprotein 1, glycoprotein 2, and RdRp.

#### Iflaviridae

**Figure 6:**
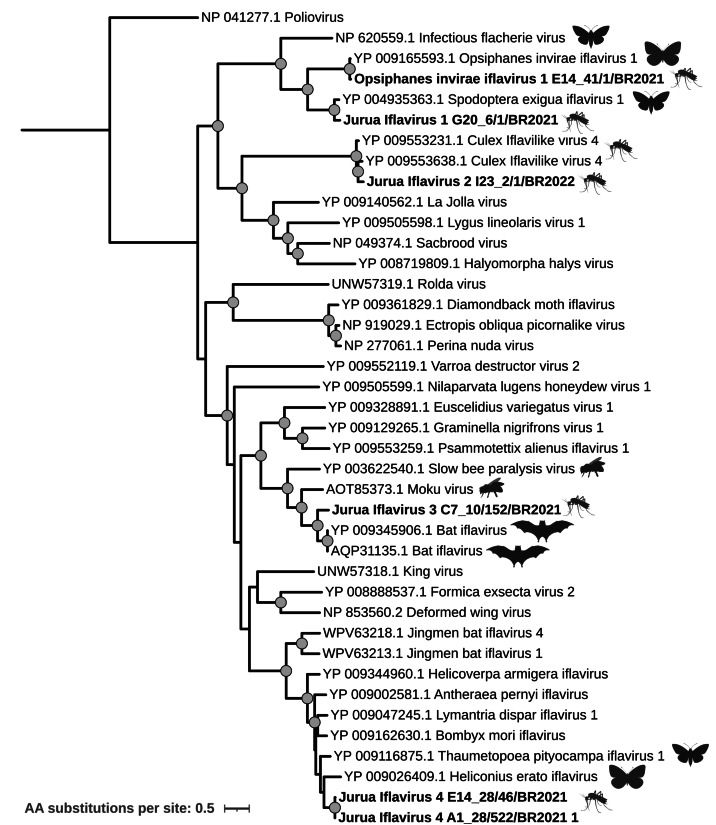
Maximum likelihood phylogeny of a partial (2,066 sites) iflavirus polyprotein sequence. Sequences obtained in this study are identified in bold. Small icons represent the known hosts for the closest related viruses to the sequences obtained in this study. Bootstrap values greater than 85 are shown with circles.

A total of eleven contigs from seven different pools were identified as belonging to the *Iflaviridae* family. Two complete vMAGs, each corresponding to a single polyprotein ORF, were recovered from pools E14_41 (strain E14_41/1/BR2021, 10,155 bp) and G20_6 (strain G20_6/1/BR2021, 10,239 bp). Strain E14/1/BR2021 was classified as Opsiphanes invirae iflavirus, with 97.0% amino acid identity across the polyprotein. In contrast, strain G20_6/1/BR2021 shared 83.4% amino acid identity with its best match (Spodoptera exigua iflavirus) and was therefore tentatively named Jurua iflavirus 1. Phylogenetic analysis using representative sequences from the *Iflaviridae* family grouped these genomes within a well-supported monophyletic clade that also included infectious flacherie virus (Fig. 6). Except for the sequences obtained in this study (associated with mosquito hosts), all the previously reported viral strains from this clade were associated with lepidopteran hosts.

In addition to the complete genomes, four partial polyprotein sequences were also included in the phylogenetic analysis. One of them, recovered from pool I23_2 (strain I23_2/1/BR2022), shared 87.2% amino acid identity with culex iflavilike virus 4 (Fig. 6). Although closely related, the level of divergence observed at the polyprotein level suggests that this strain represents a distinct virus, which we tentatively named Jurua iflavirus 2.

Another partial polyprotein sequence, obtained from pool C7_10, showed 74.2% amino acid identity to two Bat iflavirus genomes and clustered in a clade that also includes Moku virus and Slow bee paralysis virus, both originally isolated from hymenopteran hosts (Fig. 6). This virus was named Jurua iflavirus 3.

Sequences from pools E14_28 and A1_28 grouped closely, with 90.1% amino acid similarity between them, and they both had *Heliconius erato* iflavirus as their best hit, showing 78.2% and 79.1% amino acid identity, respectively. These two sequences represent strains from the same virus that we tentatively named Jurua Iflavirus 4. The clade where these strains are grouped is composed of viral strains isolated from lepidopteran hosts (Fig. 6).

#### Togaviridae

**Figure 7:**
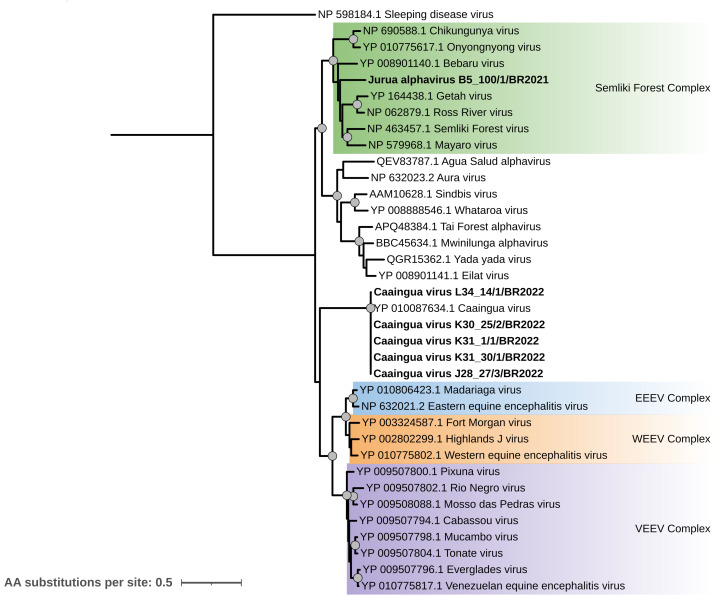
Maximum likelihood phylogenetic tree for the RdRp protein sequence from the *Togaviridae* family. Sequences obtained in this study are shown in bold. Circles on nodes indicate bootstrap values ≥85. Colors represent previously described major clades identified for this family: Western Equine Encephalitis virus (WEEV), Semliki Forest virus, Eastern Equine Encephalitis virus (EEEV), and Venezuelan Equine Encephalitis virus (VEEV) Complexes.

Thirteen contigs from six different pools were identified as belonging to the family *Togaviridae*. Five complete vMAGs and one complete nsp4 protein sequence were obtained (Table 1) and used to perform a phylogenetic tree with representative sequences of the family. Five of these sequences (from pools L34_14, K30_25, K31_1, K31_30, and J28_27) correspond to strains of the previously reported Caaingua virus (≥98% RdRp amino acid identity), isolated in Southern Brazil in 2017 (Tschá et al., 2019). The Caaingua virus strains form a monophyletic clade with high statistical support, and do not cluster with any of the previously described complexes for this family (Fig. 7).

The remaining vMAG for this family (from pool B5_100) clusters within the Semliki Forest Complex with high bootstrap values, grouping with well-known arboviruses like Chikungunya, Getah, Ross River, Semliki Forest, and Mayaro viruses (Fig. 7). Based on amino acid identity, the closest strain for this genome was Semliki Forest virus, showing 70.9% amino acid identity with the RdRp protein sequence. Thus, this genome was classified as a novel virus tentatively named Jurua alphavirus.

#### Flaviviridae

**Figure 8:**
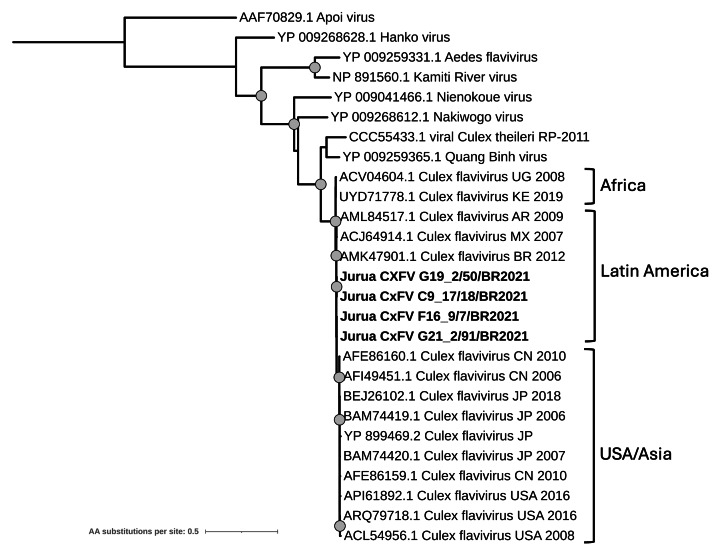
Maximum likelihood phylogenetic tree for the RdRp protein sequence from Culex flavivirus and closely related species. Sequences obtained in this study are shown in bold. Circles on nodes indicate bootstrap values ≥85. Geographic locations for the CxFV strains are indicated.

A total of thirty-three contigs from eleven mosquito pools were associated with the *Flaviviridae* family, all showing more than 90% amino acid identity with the RdRp protein of Culex flavivirus (CxFV) (Fig. S3). Phylogenetic analysis was conducted using sequences from three pools, which grouped with previously reported CxFV strains isolated in Latin America, including one strain collected in Brazil in 2012 (Fig. 8). The phylogenetic tree structure reflects a geographic pattern, clustering the different CxFV strains into three main clades: Africa, Latin America, and a group containing sequences from USA and Asia. This aligns with the genotypic classifications described in previous studies (Bittar et al., 2016; Miranda et al., 2019).

#### Mesoniviridae

**Figure 9:**
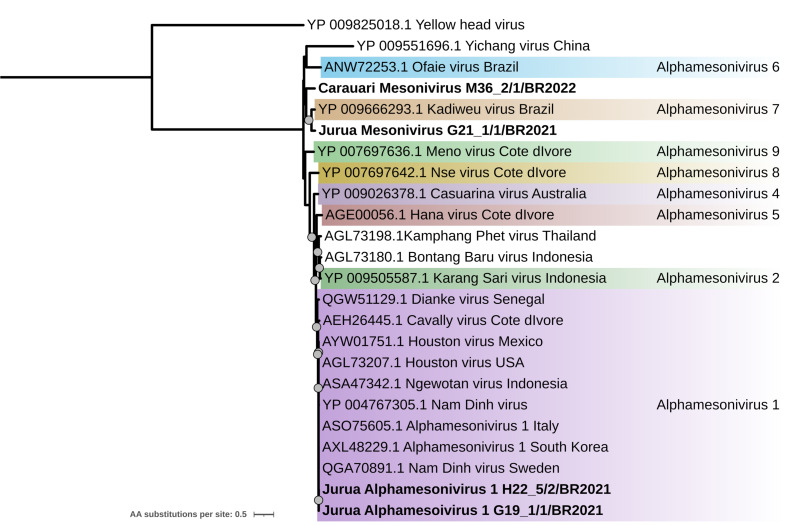
Maximum likelihood phylogeny of the complete Mesonivirus ORF1b protein sequence. Sequences obtained in this study are identified in bold. Bootstrap values greater than 85 are shown.

Eleven contigs from seven different pools were identified as belonging to the *Mesoniviridae* family. Three contigs corresponded to complete vMAGs (pools G19_1, G21_1, and M36_2). Partial RdRp sequences for four strains were employed to construct a phylogenetic tree using previously reported Alphamesonivirus viral strain sequences. The phylogenetic tree placed strains M36_2/1/BR2021 and G21_1/1/BR2021 within branches closely related to Kadiweu virus (isolated in Brazil in 2010 and also called Alphamesonivirus 7 according to Newton et al. (2020), with 75.3% and 87.6% identity, respectively. These two tentative novel viruses were named Carauari mesonivirus (strain M36_2/1/BR2021) and Jurua mesonivirus (strain G21_1/1/BR2021) (Fig. 9).

The other two strains obtained for this family, H22_5/2/BR2021 and G19_1/1/BR2021, presented identical amino acid sequences and were grouped within a clade composed of strains of Alphamesonivirus 1 which have been isolated worldwide (Fig. 9). Amino acid identity values between all the sequences from the *Mesoniviridae* dataset are found in Fig. S4.

#### Totiviridae

**Figure 10:**
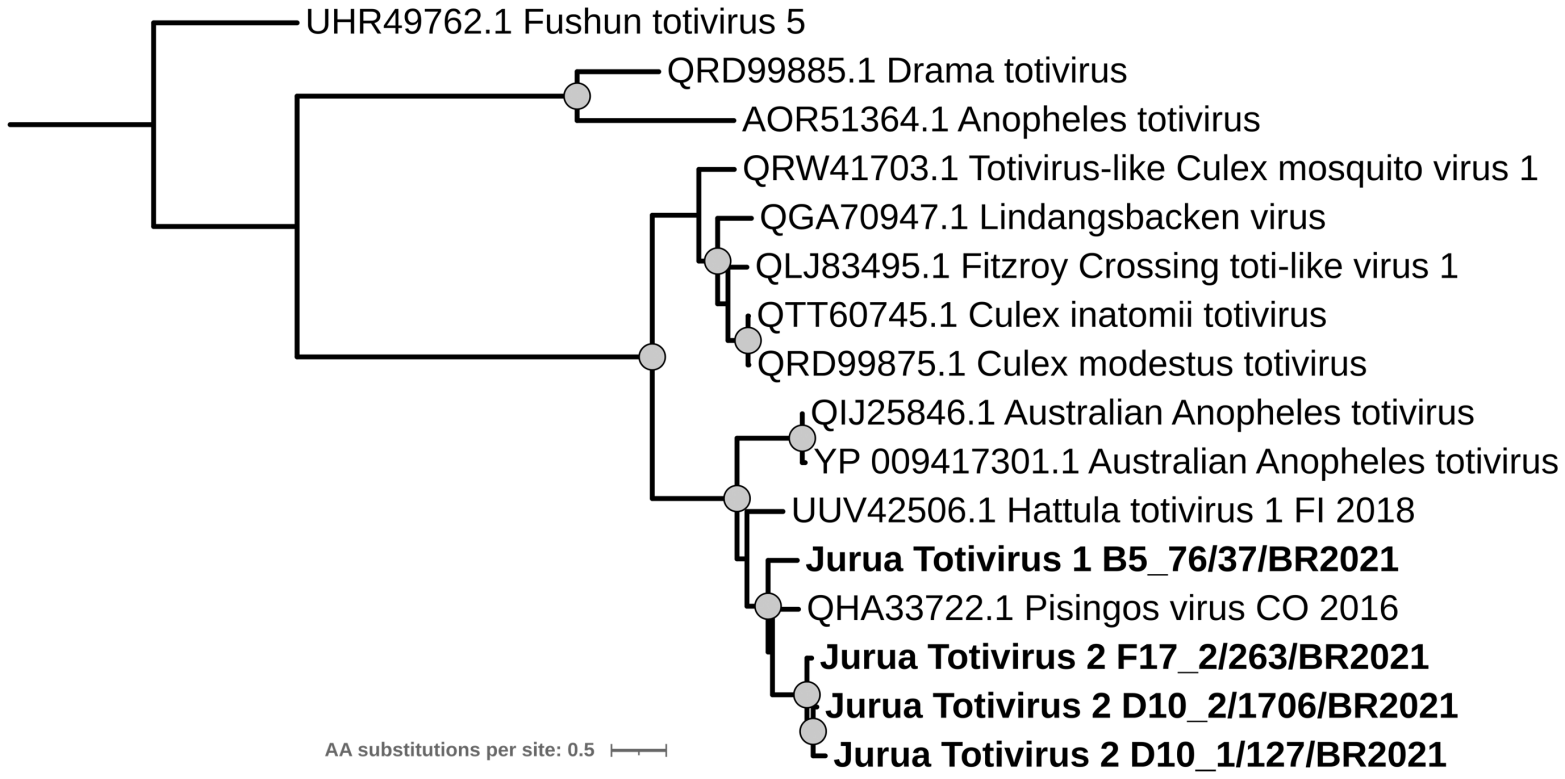
Maximum likelihood phylogeny based on the partial (553 sites) RdRp protein sequence of Totivirus strains isolated from mosquitoes. Sequences obtained in this study are identified in bold. Bootstrap values greater than 85 are indicated with a circle.

Five contigs from five different pools were associated with the *Totiviridae* family, all of them with Australian Anopheles totivirus as the best hit according to DIAMOND results. Four RdRp protein sequence were retrieved from the contigs sequences and used to generate a phylogenetic tree with Totivirus strains isolated from mosquito species retrieved from the GenBank database. Phylogenetic tree grouped the sequences herein obtained and Australian Anopheles virus sequences together in a clade with big statistical support, along with Hattula virus 1 and Pisingos virus, isolated from Finland and Colombia, respectively (Fig. 10). Phylogenetic tree, as well as amino acid distance analysis, showed that the sequences obtained in this study correspond to two tentative novel viruses named Jurua totivirus 1–2 and closely related to Pisingos virus (Fig. 10, Fig. S5).

## Discussion

This study provides a comprehensive analysis of the RNA viral communities associated with wild-caught adult female mosquito populations from the Jurua River region in the Amazon Basin. Leveraging one of the largest mosquito pool datasets to date, we identified RNA viruses from eighteen families, including twenty-one complete or nearly complete viral metagenomic assembled genomes (vMAGs) with high completeness (≥95% completeness according to CheckV) and remarkable diversity. Our pooling strategy prioritized broad geographic coverage and viral discovery across sites, enabling the detection and genetic characterization of multiple viral taxa circulating in this understudied region. However, because pools combine multiple, often mixed-species individuals, our approach cannot resolve which viruses co-occur within individual mosquitoes or specific host species. Despite this limitation, these findings represent a significant contribution to understanding the genetic diversity of RNA viruses circulating in this biodiverse and understudied region, while also providing insights into viral evolution and ecological dynamics.

At a genome composition level, previous virome survey studies have stated that positive-sense single-stranded RNA families such as *Flaviviridae*, *Mesoniviridae*, and *Iflaviridae* are among the most abundant and widespread viruses in mosquitoes (Chen et al., 2024; Moonen et al., 2023; Morel et al., 2024). While these families are not the most prevalent or abundant in our samples, multiple species of known and tentatively novel viruses belonging to these families were identified in our data, consistent with earlier work. At the same time, we found that negative-sense RNA families make a particularly strong contribution to viral diversity in our system. *Phasmaviridae* and *Phenuiviridae* are both highly prevalent across pools and together account for a large fraction of the viral species recovered. This pattern suggests that in the Amazonian rainforest mosquitoes, (-)ssRNA viruses can be major contributors to species richness alongside the positive-sense families.

The contrast with many published studies, where (+)ssRNA viruses are often reported as dominant, can be explained by factors related to differences in sampling (*e.g.*, ecosystem, host species). Most virome work to date has focused on recognized vector genera such *Aedes* and *Culex* and on sites with substantial human activity, frequently as part of arbovirus surveillance programs in urban or peridomestic environments (Hollingsworth et al., 2023; Liu et al., 2023; Moonen et al., 2023). Because mosquito virome composition is strongly structured by host species (Liu et al., 2023; Thongsripong et al., 2021; Wu et al., 2023), the predominance of (+)ssRNA families in these studies may partly reflect the ecology of a small set of well-studied vector species. In contrast, our study targets mixed mosquito assemblages from remote Amazonian rainforest, where mosquito diversity and viromes remain poorly characterized.

Moreover, a recent study on sylvatic mosquitoes in Brazil supports this view. Metatranscriptomic surveys of forest-dwelling mosquitoes from Northeast Brazil and the Cerrado biome revealed highly diverse viromes with a more balanced composition between positive and negative sense RNA viruses (Da Silva et al., 2024). Viral species belonging to families like *Phasmaviridae* (the most prevalent family observed in this study) and *Phenuiviridae* were described as part of the main components of the virome in species like *Coquillettidia hermanoi* and *Limatus durhamii*, which are rarely included in urban mosquito virome work. Together with our findings, these results suggest that (-)ssRNA viruses can be major contributors to diversity in sylvatic mosquito communities, even though they are less visible in datasets dominated by urban vector species.

### Expanding the knowledge of *Bunyaviricetes* in Amazonia

The recently elevated class *Bunyaviricetes* includes hundreds of segmented RNA viruses, many of which are pathogenic to humans, animals, plants, and fungi (Kuhn et al., 2024). In this study, we classified vMAGs into three families within this class: *Phasmaviridae*, *Phenuiviridae*, and *Perybunyaviridae*. Across the analyzed pools, *Phasmaviridae* emerged as the most prevalent family, represented by members of the genera *Orthophasmavirus* and *Jonvirus*. Within the *Orthophasmavirus* genus, a substantial number of vMAGs were closely related to Coredo virus, represented by one strain isolated from *Mansonia titillans* mosquitoes in Colombia in 2016. Using the ICTV-defined threshold of 95% amino acid identity in the RdRp amino acid sequence for species differentiation, our results suggest the presence of a highly diverse array of Coredo-like viruses in the Jurua region, likely representing multiple unreported species sharing a common ancestor (Fig. 3). These results support the need for continued study of this viral group, which appears to be an integral part of the local mosquito virome.

In addition to Coredo-like viruses, two strains within the *Orthophasmavirus* genus were identified: strains B6_22/775/BR2021 and J27_25/5/BR2022. These sequences form a well-supported monophyletic clade with other viruses isolated from *Anopheles* mosquitoes, including Anopheles triannulatus orthophasmavirus, previously reported in the Amazon (Scarpassa et al., 2019), and *Anopheles stephensis* orthophasmavirus, reported in Japan (Matsumura et al., 2024). While we cannot confirm the specific mosquito hosts of the newly identified viral strains, the clustering of these sequences with previously reported viruses associated with *Anopheles* species suggests the potential for similar host associations.

Further, our discovery of a new *Jonvirus* strain marks the first report of this genus outside Africa (Hermanns et al., 2023; Marklewitz et al., 2015). This finding suggests a broader geographical distribution for *Jonvirus* and underscores the utility of HTS for uncovering the evolutionary relationships and diversity of the *Phasmaviridae* family.

The detection of viral strains from the *Phenuiviridae* family revealed representatives from the genera *Mobuvirus* and *Goukovirus* (Fig. 4). These genera consist of insect-specific viruses (Sasaya et al., 2023), and the sequences identified in this study represent two tentative novel viruses closely related to Narangue virus and Cumuto virus, previously isolated from *Mansonia* and *Culex* mosquitoes, respectively (Auguste et al., 2014). The distribution of these viruses in tropical regions such as Colombia and Trinidad aligns with the environmental characteristics of the Amazon, highlighting the region’s unexplored viral diversity and the need for further exploration.

A putative complete set of genomic segments from the *Perybunyaviridae* family was obtained from pool F17_5 and assigned to the *Gamboa* virus complex within the genus *Orthobunyavirus*. The L segment showed sequence identity levels to Alajuela virus above the species demarcation criteria from ICTV, indicating the presence of this species in the pool. While the M and S segments also had Alajuela virus as their best BLASTX hit, these proteins are less conserved and no formal ICTV thresholds are defined for them. Because these data derived from pooled mosquitoes, we cannot exclude the possibility that the three segments originate from more than one closely related Gamboa complex virus (for example through reassorment or co-infection). We therefore report the presence of Alajuela virus based on the L segment and interpret the associated M and S segments as most closely related to Alajuela virus.

Members of the *Gamboa* serogroup, including *Alajuela virus*, have been isolated mainly from the avian-feeding mosquito *Aedeomyia squamipennis* across several Neotropical countries like Panama, Ecuador, Argentina, Suriname, and Brazil (Eastwood et al., 2016; Nunes et al., 2014). Transovarial transmission of Gamboa virus in *A. squamipennis* has also been demonstrated, indicating that this mosquito species can act as a maintenance host for Gamboa-serogroup viruses in nature (Dutary et al., 1989). While not linked to human disease, monitoring the genetic diversity of such viruses remains critical, as recombination events in *Orthobunyavirus* strains have led to outbreaks of pathogenic viruses, such as the recent Oropouche virus outbreak in South America (Zhang et al., 2024).

Although evidence of *Nairoviridae* was found (another family belonging to the Bunyaviricetes order), all identified contigs matched a single hypothetical protein unique to the genus *Ocetevirus* (Käfer et al., 2019) and lacked the hallmark RdRp ORF expected in this family. These contigs were later resolved as mosquito genes once we expanded the search to broader databases, leading us to remove these contigs from the viral catalog. Our re-evaluation of the putative *Nairoviridae* signal illustrates how easily host-derived sequences can masquerade as viral when annotations rely heavily on poorly characterized “hypothetical proteins”. Beyond the specific case of *Nairoviridae*, this highlights how gaps and misannotations in both viral and host genome resources can distort perceived community composition if not carefully checked. More generally, it underscores the need for conservative, multi-step annotation strategies, combining viral-focused databases with comprehensive resources such as nr, considering genome architecture and hallmark genes, and treating isolated hits to hypothetical proteins with caution, to avoid overinterpreting spurious signals in metagenomic virome studies.

### Insights into *Mononegavirales* diversity

Within the *Mononegavirales* order, two families were identified: *Rhabdoviridae* and *Xinmoviridae*. *Rhabdoviridae* was the second most prevalent family, with sixteen new vMAGs corresponding to Merida virus. This virus was originally reported in Mexico in 2016 (Charles et al., 2016) and later identified in the USA (Sadeghi et al., 2018). To date, Merida virus has been reported in mosquitoes from four continents and in multiple mosquito genera, including *Culex*, *Aedes,* and *Haemagogus* (Moonen et al., 2023). Our data extends its known distribution to the Western Amazon, representing the first report of Merida virus in South America. The high prevalence of Merida virus in our pools is consistent with previous surveillance studies in which rhabdoviruses, including Merida-like viruses, are among the most frequently detected and widespread mosquito-associated viruses (Moonen et al., 2023; Morel et al., 2024). While Merida virus had not been reported in South America before this study, sequences with lower amino acid identity values (51%) to *Merida virus* were previously reported in the Amazon region (Da Silva Neves et al., 2021), suggesting the circulation of unreported closely related species in the region that warrants further investigation.

Phylogenetic analysis revealed three distinct clades, likely reflecting the geographic separation between strains from Asia, Europe, and the Americas. For generalist mosquito-associated viruses such as Merida virus, the ability to infect multiple mosquito species has been proposed as one explanation for their global distribution, along with co-radiation with culicids during their evolution and spread (Morel et al., 2024). Furthermore, other genera within *Rhabdoviridae*, such as *Nucleorhabdovirus* and *Cythorhabdovirus*, are known to infect plants and animals while using arthropods as vectors, facilitating the spread of these viruses (Whitfield et al., 2018) and demonstrating that multi-host life cycles can occur within this family. Whether Merida virus and related mosquito-associated rhabdoviruses share such complex ecologies is currently unclear, and more comprehensive screening of mosquitoes and potential non-mosquito hosts will be needed to clarify their evolution and host range.

### *Iflaviridae*: host associations and ecological links

The *Iflaviridae* family comprises viruses with a non-segmented, (+)ssRNA genome. To date, and according to the ICTV, Iflaviruses exclusively infect arthropod hosts, with the majority reported in insects. While this family currently lacks formal genus-level classifications, phylogenetic analyses have consistently revealed the presence of distinct clades with strong statistical support, suggesting shared ancestry among viral strains (Valles et al., 2017).

The viral sequences identified in this study fall into four of these distinct clades and are closely related to viruses previously isolated from various insect orders, including Diptera (*e.g.*, flies) and Lepidoptera (*e.g.*, moths and butterflies). This is consistent with earlier studies indicating that *Iflaviridae* evolution does not follow a strict host-based pattern, as each clade contains viruses infecting hosts from different insect groups (Valles et al., 2017).

A particularly notable result is the close relationship between one of our sequences (a tentatively new virus named Jurua iflavirus 3) and Bat iflavirus (Fig. 6). Viral strains from the *Iflaviridae* family, such as Bat iflavirus and King virus, have been associated with bats (He et al., 2013; Juergens, Huckabee & Greninger, 2022; Yinda et al., 2017). However, these strains have been isolated from either bat guano or intestinal contents, making it difficult to distinguish between true vertebrate infection and viruses derived from ingested arthropods. Thus, the most plausible explanation is that Jurua iflavirus 3 represents an ISV that can also be detected in bat-associated samples *via* dietary or environmental exposure, rather than an arbovirus lifestyle. Our phylogenetic results therefore point to ecological links between mosquitoes and insectivorous vertebrates. Further studies are needed in order to determine whether any iflaviruses are capable of replicating in vertebrate hosts.

### *Togaviridae* and *Flaviviridae*: implications for public health and virus evolution

The *Togaviridae* family consists of non-segmented, (+)ssRNA viruses currently classified into a single genus, *Alphavirus* (Strauss & Strauss, 1994). In this study, we identified and characterized the complete genome of a novel alphavirus, Jurua alphavirus (11Kb genome length), which was classified within the Semliki Forest Complex (Fig. 7), a major clade recognized by the ICTV. Notably, insect-specific alphaviruses included in the phylogenetic analysis like Eilat virus, Aqua salad alphavirus, and Yada Yada virus, form a monophyletic clade with high bootstrap values, supporting the evidence that Jurua alphavirus is more evolutionary related to the arboviruses from the Semliki Forest Complex rather than to insect-specific viruses from the *Togaviridae* family. The Semliki Forest Complex includes Old-World alphaviruses known to cause diseases in humans and animals, such as Chikungunya virus and Mayaro virus. These viruses are associated with similar symptoms like fever, headache, and arthralgia (Henss et al., 2019). While Brazil is one of the countries with the highest recency and frequency of Chikungunya cases (Grabenstein & Tomar, 2023), epidemics of Mayaro fever have been reported in several regions of Latin America, including the Amazon Basin (Henss et al., 2019; Mourão et al., 2012). The identification of Jurua alphavirus, a previously unrecognized virus within this medically significant clade, underscores the need for further investigation into its pathogenic potential, host range, and prevalence in the region. Given the history of emerging alphaviruses causing epidemic outbreaks in the region (Azar et al., 2020; Resck et al., 2024), enhanced surveillance efforts are warranted to monitor the presence and possible spillover risks associated with this virus.

Additionally, this study provides four complete genomes of Caaingua virus (CAAV), an insect-specific alphavirus isolated from mosquito pools of *Culex* sp. in Southern Brazil in 2019 (Tschá et al., 2019). Phylogenetic analysis indicates that CAAV strains form a monophyletic clade that is basal to the New World encephalitic alphavirus side (WEE/VEE complexes, see Fig. 7) without clustering with any currently defined complex. This topology is consistent with broader alphavirus phylogenies in more recent work (Silva et al., 2022). These findings highlight the unexplored genetic diversity within the *Togaviridae* family and suggest that CAAV-like viruses may present a distinct evolutionary history from the better-studied Old World and New World Alphavirus complexes*.* Our recovery of multiple CAAV strains from Amazonian mosquito pools extends the known geographic and ecological range of this lineage and underscores that much of the basal diversity of Alphaviruses remains unsampled in sylvatic mosquito communities.

The *Flaviviridae* family, another medically significant group of (+)ssRNA viruses, includes both medically important arboviruses and Insect-Specific Flaviviruses (ISFV), which have gained particular interest for their potential interactions with pathogenic flaviviruses (Vasilakis & Tesh, 2015). Among the flaviviruses of greatest public health concern, Dengue virus, Yellow Fever virus, and Zika virus are all endemic to the Amazon region (Barreto et al., 2020; Figueiredo, 2000; Rincón et al., 2024). However, in addition to these arboviruses, ISFVs are increasingly recognized as key components of mosquito viromes, as their widespread distribution and host-restricted replication suggest they may influence the transmission dynamics of pathogenic flaviviruses (Kent, Crabtree & Miller, 2010; Talavera et al., 2018). In this study, we identified sequences corresponding to Culex Flavivirus (CxFV), an ISFV distributed worldwide.

Our findings further demonstrate the presence and repeated detection of ISFV in Amazonian mosquito populations, with sequences identified in eleven mosquito pools. The four CxFV strains characterized in this study contribute to a better understanding of the genetic diversity and geographic distribution of ISFVs. Additionally, our phylogenetic analysis supports the previously proposed genotypic classification of CxFV, which appears to follow a geographic clustering pattern (Miranda et al., 2019). Given the potential role of ISFVs in influencing the replication and transmission of medically important flaviviruses, these findings emphasize the importance of continued surveillance in the Amazon Basin to monitor co-circulating arboviruses and ISFVs and to assess their ecological interactions.

### *Mesoniviridae*: expanding the known genetic diversity of an emerging insect-specific family

The *Mesoniviridae* family, classified within the *Nidovirales* order, consists of (+)ssRNA viruses and represents the first family in the order reported to infect insects (Zirkel et al., 2013). This makes *Mesoniviridae* particularly relevant from an evolutionary perspective, as its origins may provide insights into the broader evolutionary history of *Nidovirales*, which includes viral families of significant concern such as *Coronaviridae*. *Mesoniviridae* has been described as a missing link in the transition from small to large nidoviruses, representing an intermediate step in the genome expansion and complexity within the order (Nga et al., 2011). The study of this family is therefore critical to understanding the evolutionary pressures and mechanisms that may have led to the emergence of larger nidoviruses, including coronaviruses.

Since it was first described, the *Mesoniviridae* family has rapidly expanded, with new species being identified and classification systems continuously refined (Morais et al., 2022). In this study, we identified four new complete mesonivirus genomes (∼20Kb), two of which correspond to strains of Alphamesonivirus 1, the most frequently isolated mesonivirus to date. Additionally, we describe two novel mesoniviruses, Carauari mesonivirus and Jurua mesonivirus, both closely related to Kadiweu virus (KADV), also named Alphamesonivirus 7, originally isolated from the High Pantanal region in Brazil (Maia et al., 2019). These findings expand the known diversity of mesoniviruses in Brazil (which also includes Ofaie virus), demonstrating the presence of a rich but largely unexplored mesonivirus diversity in South America.

### *Totiviridae*: emerging diversity and potential role in mosquito viromes

The *Totiviridae* family comprises dsRNA viruses originally known to infect fungi, protozoa, and plants (Ghabrial & Nibert, 2009). However, an increasing number of totiviruses have also been identified in insects, including mosquitoes (Fauver et al., 2016; Truong Nguyen et al., 2022; Zhai et al., 2010). Despite their widespread detection across diverse species and global distribution, mosquito-associated totiviruses remain unclassified by the ICTV, so their taxonomic placement remains incomplete. Moreover, the majority of them are known only from metagenomic data, leaving their ecology and host interactions poorly understood.

In this study, we identified two novel mosquito-associated totiviruses, Jurua totivirus 1 and Jurua totivirus 2, which are closely related to Pisingus virus, a totivirus isolated from *Psorophora funiculus* in Colombia in 2016. Our findings add to the growing catalog of mosquito-associated totiviruses and extend the known geographic range of this lineage into the Western Amazon. This pattern is consistent with surveillance studies that report frequent detection and high prevalence of totiviruses in mosquito populations (Coatsworth et al., 2022; Truong Nguyen et al., 2022). However, further analyses will be needed to clarify their host range and potential ecological roles in mosquito biology and virus-host interactions.

## Conclusions

This study represents one of the most extensive viral surveillance efforts conducted in the Amazon Basin to date, analyzing over 200 mosquito pools across a vast and biodiverse region. By leveraging HTS and *de novo* assembly, we uncovered an unprecedented diversity of RNA viruses, expanding the known virome of mosquitoes in the Amazon. The identification of multiple novel viral species, alongside new records of viruses in the region, underscores the immense, yet largely unexplored, viral diversity circulating in these ecosystems. These findings highlight the critical importance of large-scale surveillance studies in biodiverse and ecologically dynamic areas, not only to advance our understanding of virus-mosquito interactions but also to provide essential insights into viral evolution and potential emerging viruses that could be important for human and animal health.

## Supplemental Information

10.7717/peerj.20880/supp-1 Supplemental Information 1 Sampling sites where mosquito pools were collected Coordinates for each sampling site are shown, as well as year of collection, number of pools, and total number of individuals

10.7717/peerj.20880/supp-2 Supplemental Information 2 Summary of RNA viral genomes and RdRp contigs recovered from mosquito pools Viral metagenome-assembled genomes (vMAGs) and RdRp-containing contigs identified in this study. For each sequence, we report the provisional virus name, best BLASTX hit (virus and protein accession), amino-acid identity (%), viral family, mosquito pool ID, strain ID, isolation year, GenBank accession, contig length (bp), segment (where applicable), expected set of genes, genes observed on the contig, CheckV completeness estimate (%), and the final completeness category (e.g. complete CDS, partial RdRp CDS, complete CDS for all three segments).
An asterisk (* ) after the virus name denotes tentatively novel viruses described in this study. A double asterisk (**) indicates complete genomes recovered.

10.7717/peerj.20880/supp-3 Supplemental Information 3 Sequencing result details for each pool Number of paired-reads before and after quality check are indicated, along with file names for the fastq files and BioSample IDs.

10.7717/peerj.20880/supp-4 Supplemental Information 4 Sequence clustering of Coredo-like virus sequences identified in this study Clustering was performed using CD-HIT with a 98% amino acid identity threshold. For each cluster, the representative sequence used for phylogenetic analysis is indicated, along with the sequences comprising each cluster and their amino acid identity to the representative.

10.7717/peerj.20880/supp-5 Supplemental Information 5 Sequence clustering of Jurua Merida virus sequences identified in this study Clustering was performed using CD-HIT with a 98% amino acid identity threshold. The representative sequence used for phylogenetic analysis is indicated, along with the sequences comprising the cluster and their amino acid identity to the representative.

10.7717/peerj.20880/supp-6 Supplemental Information 6 Amino acid identity matrix based on the RdRp protein sequence of Merida-like viruses identified in this study and reference sequences Color shading represents the degree of identity, with darker blue indicating higher similarity.

10.7717/peerj.20880/supp-7 Supplemental Information 7 Amino acid identity matrix based on the RdRp protein sequence of strains from the *Madalivirus* genus Color shading represents the degree of identity, with darker blue indicating higher similarity.

10.7717/peerj.20880/supp-8 Supplemental Information 8 Amino acid identity matrix based on the RdRp protein sequence of the Culex flavivirus strains employed in this study Color shading represents the degree of identity, with darker blue indicating higher similarity.

10.7717/peerj.20880/supp-9 Supplemental Information 9 Amino acid identity matrix based on the ORF1b protein sequence of the Mesonivirus strains employed in this study Color shading represents the degree of identity, with darker blue indicating higher similarity.

10.7717/peerj.20880/supp-10 Supplemental Information 10 Amino acid identity matrix based on the RdRp protein sequence of the Totivirus strains employed in this study Color shading represents the degree of identity, with darker blue indicating higher similarity.

10.7717/peerj.20880/supp-11 Supplemental Information 11 Viral genomes obtained in this study Viral genomes (complete and partial) and their respective annotations as they were submitted to the GenBank. Accession numbers assigned to the sequences can be found in Table 1. Most of these sequences are not publicly available yet

## Additional Information and Declarations

### Field Study Permissions

The following information was supplied relating to field study approvals (i.e., approving body and any reference numbers): Chico Mendes Institute for Biodiversity Conservation, Ministry of the Environment, Federal Government of Brazil.

### Data Availability

The following information was supplied regarding data availability: The 65 viral sequences are available in the Genbank database (Table 1). Raw sequencing data is available at the NCBI repository under BioProject PRJNA1223432.
