# Supplementary material for: Large-scale metagenomic surveillance study expands the known diversity of RNA viruses in mosquito populations from the Amazon Basin
Source: PeerJ. 2026 Mar 11;14:e20880. doi: 10.7717/peerj.20880 (PMC12988728; doi:10.7717/peerj.20880)
Supplement: Supplemental Information 1 — Coordinates for each sampling site are shown, as well as year of collection, number of pools, and total number of individuals [file peerj-14-20880-s001.pdf]

Sampling Site	Latitude	Longitude	Collection year	Total Pools	Total individuals
A1	-4.526067	-66.61763	2021	6	300
A2	-4.521033	-66.62268	2021	6	300
A3	-4.515583	-66.6242	2021	6	300
B4	-4.590917	-66.64247	2021	6	300
B5	-4.58915	-66.64818	2021	7	350
B6	-4.589567	-66.63717	2021	4	200
C7	-5.056667	-67.14965	2021	6	300
C8	-5.054683	-67.15097	2021	4	200
C9	-5.04715	-67.14893	2021	6	300
D10	-5.55025	-67.48303	2021	6	300
D11	-5.538967	-67.4754	2021	7	350
D12	-5.572067	-67.50063	2021	4	200
E13	-5.474583	-67.41282	2021	6	300
E14	-5.473033	-67.4086	2021	6	300
E15	-5.484033	-67.40995	2021	6	300
F16	-4.853883	-66.88293	2021	6	300
F17	-4.8249	-66.86607	2021	7	350
F18	-4.820767	-66.85372	2021	6	300
G19	-4.8444	-66.9028	2021	4	200
G20	-4.873183	-66.90002	2021	6	300
G21	-4.77275	-66.96207	2021	6	300
H22	-4.872633	-66.89582	2021	8	400
I23	-4.740867	-66.73883	2022	6	300
I24	-4.740867	-66.73887	2022	6	300
I25	-4.74375	-66.72025	2022	6	300
J26	-4.68635	-66.68702	2022	6	300
J27	-4.6884	-66.68255	2022	6	300
J28	-4.676817	-66.69118	2022	6	300
K29	-5.17555	-67.21802	2022	6	300
K30	-5.174433	-67.21605	2022	6	300
K31	-5.178883	-67.21292	2022	6	300
L32	-5.082217	-67.02955	2022	6	300
L33	-5.084117	-67.03122	2022	6	300
L34	-5.065817	-67.14517	2022	6	300
M35	-4.934567	-66.92958	2022	2	100
M36	-4.9197	-66.92853	2022	2	100
M37	-4.934467	-66.92277	2022	6	300
