# Supplementary material for: Large-scale metagenomic surveillance study expands the known diversity of RNA viruses in mosquito populations from the Amazon Basin
Source: PeerJ. 2026 Mar 11;14:e20880. doi: 10.7717/peerj.20880 (PMC12988728; doi:10.7717/peerj.20880)
Supplement: Supplemental Information 4 — Clustering was performed using CD-HIT with a 98% amino acid identity threshold. For each cluster, the representative sequence used for phylogenetic analysis is indicated, along with the sequences comprising each cluster and their amino acid identity to the representative. [file peerj-14-20880-s004.pdf]

**Cluster's Reepresentative Strain (Length)**	**Clustered Strains**	**ORF Length**	**%Id to Rep. Sequence (aa)**
Jurua Coredo-like virus 2 D10_16/3/BR2021 (1534 aa)	C7_12/19/BR2021	815 aa	99.26
	C8_15/82/BR2021	484 aa	99.79
	D10_8/18/BR2021	720 aa	100
	E14_14/35/BR2021	1043 aa	98.27
	F16_7/34/BR2021	633 aa	100
Jurua Coredo-like virus 3 B4_59/8/BR2021 (1362 aa)	B4_96/22/BR2021	825 aa	99.76
	B5_100/3/BR2021	701 aa	100
	B5_2/984/BR2021	357 aa	99.16
	I24_20/37/BR2022	518 aa	100
	I24_2/44/BR2022	445 aa	99.78
	J26_21/216/BR2022	715 aa	99.86
	J26_9/180/BR2022	511 aa	99.8
	J27_24/85/BR2022	600 aa	99.83
Jurua Coredo-like virus 3 B4_60/13/BR2021 (1316 aa)	A3_18/51/BR2021	743 aa	99.87
	I23_2/148/BR2022	342 aa	100
	I24_5/15/BR2022	585 aa	99.66
	J26_19/389/BR2022	334 aa	100
Jurua Coredo-like virus 3 I23_87/2/BR2022 (1243 aa)	I25_31/6/BR2022	819 aa	100
	B4_82/35/BR2021	939 aa	100
Jurua Coredo-like virus 1 B5_87/7/BR2021 (1815 aa)	B5_84/7/BR2021	1706 aa	99.53
	D10_17/30/BR2021	609 aa	99.51
	D11_14/100/BR2021	427 aa	99.3
	D11_2/141/BR2021	836 aa	99.64
	D11_6/167/BR2021	397 aa	99.5
	I24_22/11/BR2022	772 aa	99.48
