# Supplementary material for: Large-scale metagenomic surveillance study expands the known diversity of RNA viruses in mosquito populations from the Amazon Basin
Source: PeerJ. 2026 Mar 11;14:e20880. doi: 10.7717/peerj.20880 (PMC12988728; doi:10.7717/peerj.20880)
Supplement: Supplemental Information 5 — Clustering was performed using CD-HIT with a 98% amino acid identity threshold. The representative sequence used for phylogenetic analysis is indicated, along with the sequences comprising the cluster and their amino acid identity to the representative. [file peerj-14-20880-s005.pdf]

**Cluster's Reepresentative Strain (Length)**	**Clustered Strains**	**ORF Length**	**%Id to Rep. Sequence (aa)**
Jurua MERDV C7_13/BR2021 (2136 aa)	Jurua MERDV C8_31/BR2021	2136 aa	99.63
	Jurua MERDV F16_1/BR2021	2136 aa	98.64
	Jurua MERDV F16_2/BR2021	2136 aa	99.77
	Jurua MERDV F16_9/BR2021	2136 aa	98.64
	Jurua MERDV G19_1/BR2021	2136 aa	99.77
	Jurua MERDV G19_2/BR2021	2136 aa	99.77
	Jurua MERDV G19_4/BR2021	2136 aa	98.67
	Jurua MERDV H22_1/BR2021	2136 aa	99.67
	Jurua MERDV H22_10/BR2021	2136 aa	99.72
	Jurua MERDV H22_12/BR2021	2136 aa	99.72
	Jurua MERDV H22_2/BR2021	2136 aa	99.77
	Jurua MERDV H22_3/BR2021	2136 aa	99.77
	Jurua MERDV H22_5/BR2021	2136 aa	99.77
	Jurua MERDV H22_8/BR2021	2136 aa	99.77
	Jurua MERDV H22_9/BR2021	2126 aa	99.87
