# Supplementary figures and images for: Large-scale metagenomic surveillance study expands the known diversity of RNA viruses in mosquito populations from the Amazon Basin
Source: PeerJ. 2026 Mar 11;14:e20880. doi: 10.7717/peerj.20880 (PMC12988728; doi:10.7717/peerj.20880)

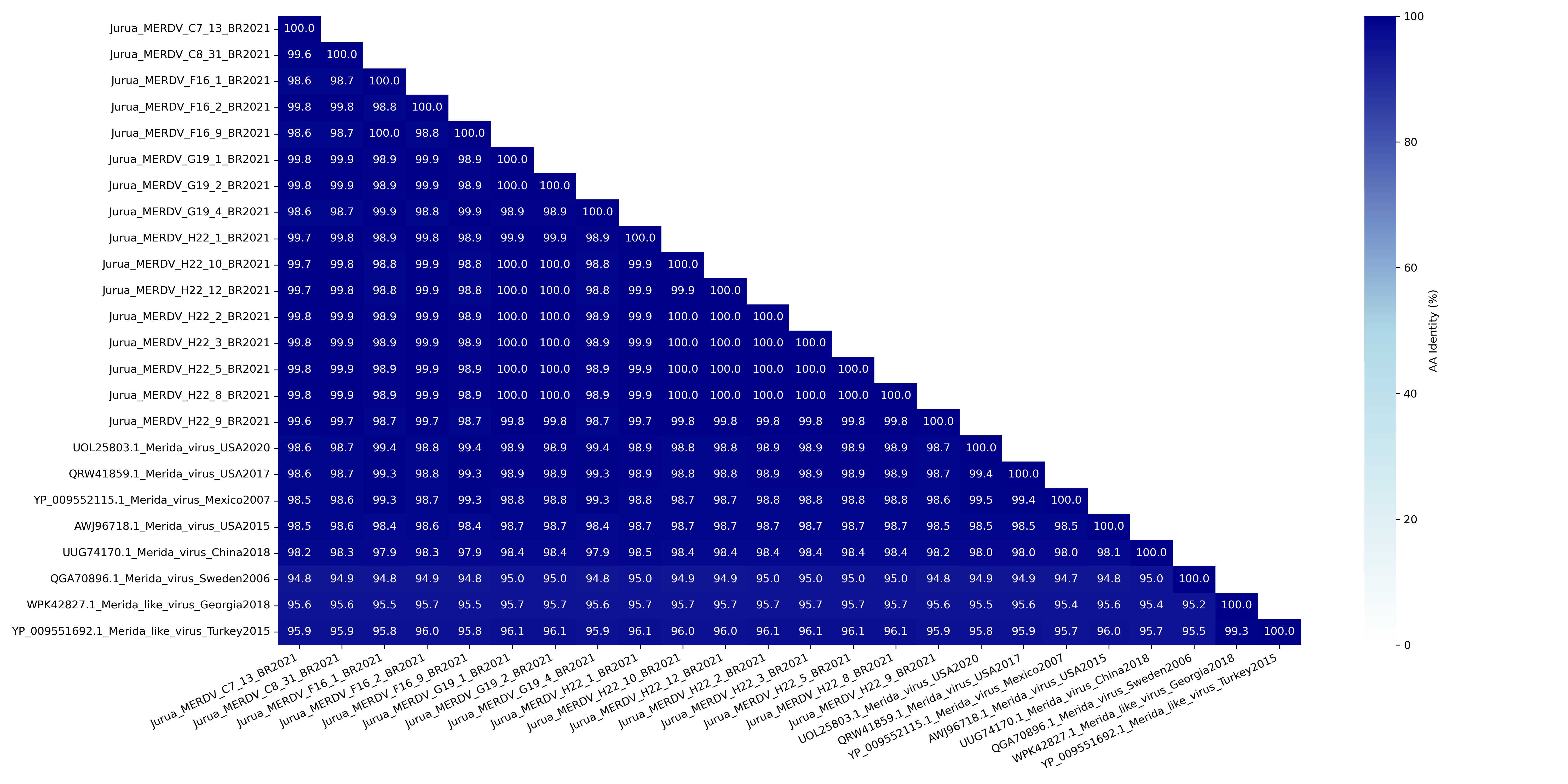

Supplement: Supplemental Information 6 — Color shading represents the degree of identity, with darker blue indicating higher similarity. [file peerj-14-20880-s006.png]

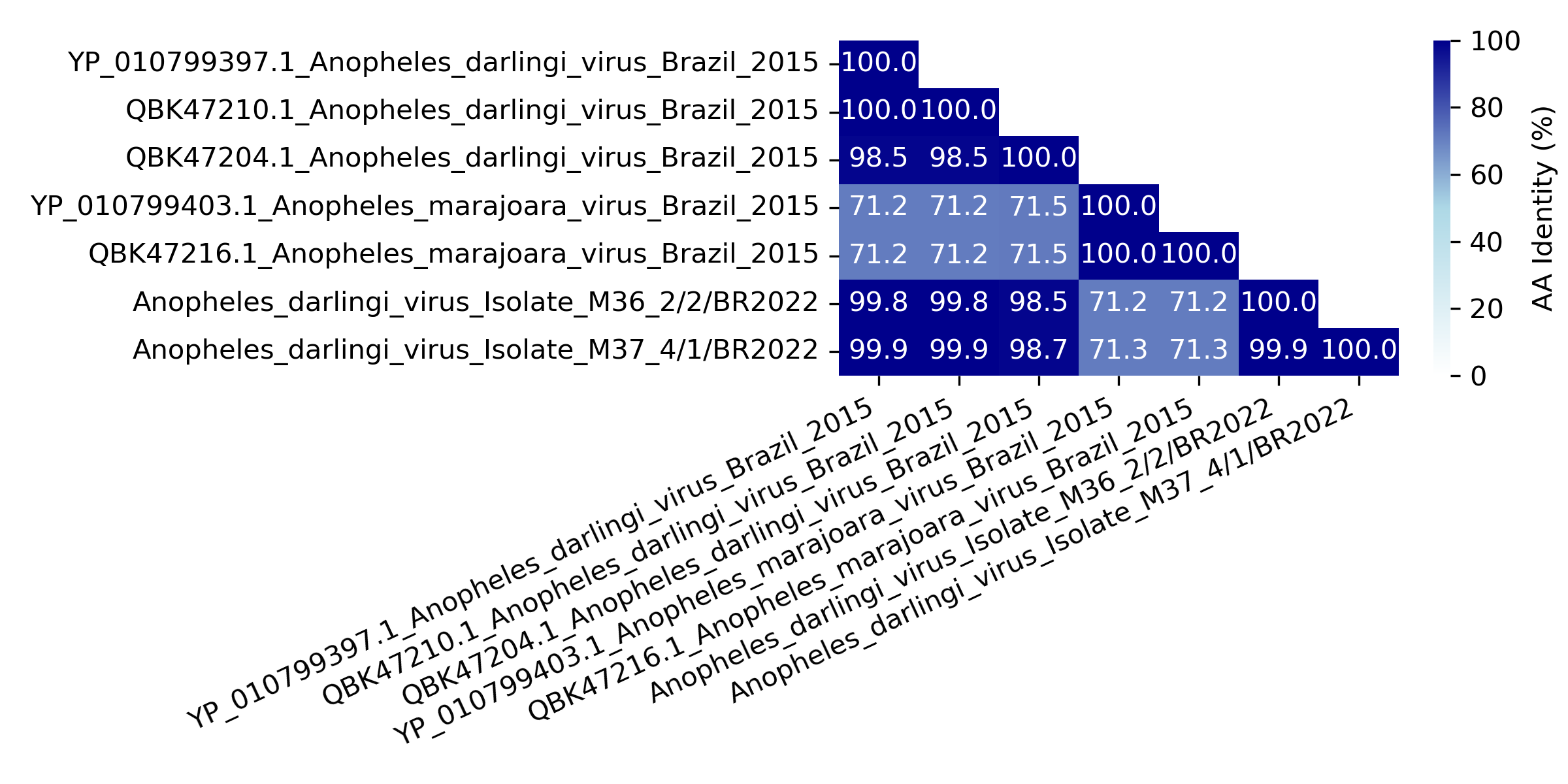

Supplement: Supplemental Information 7 — Color shading represents the degree of identity, with darker blue indicating higher similarity. [file peerj-14-20880-s007.png]

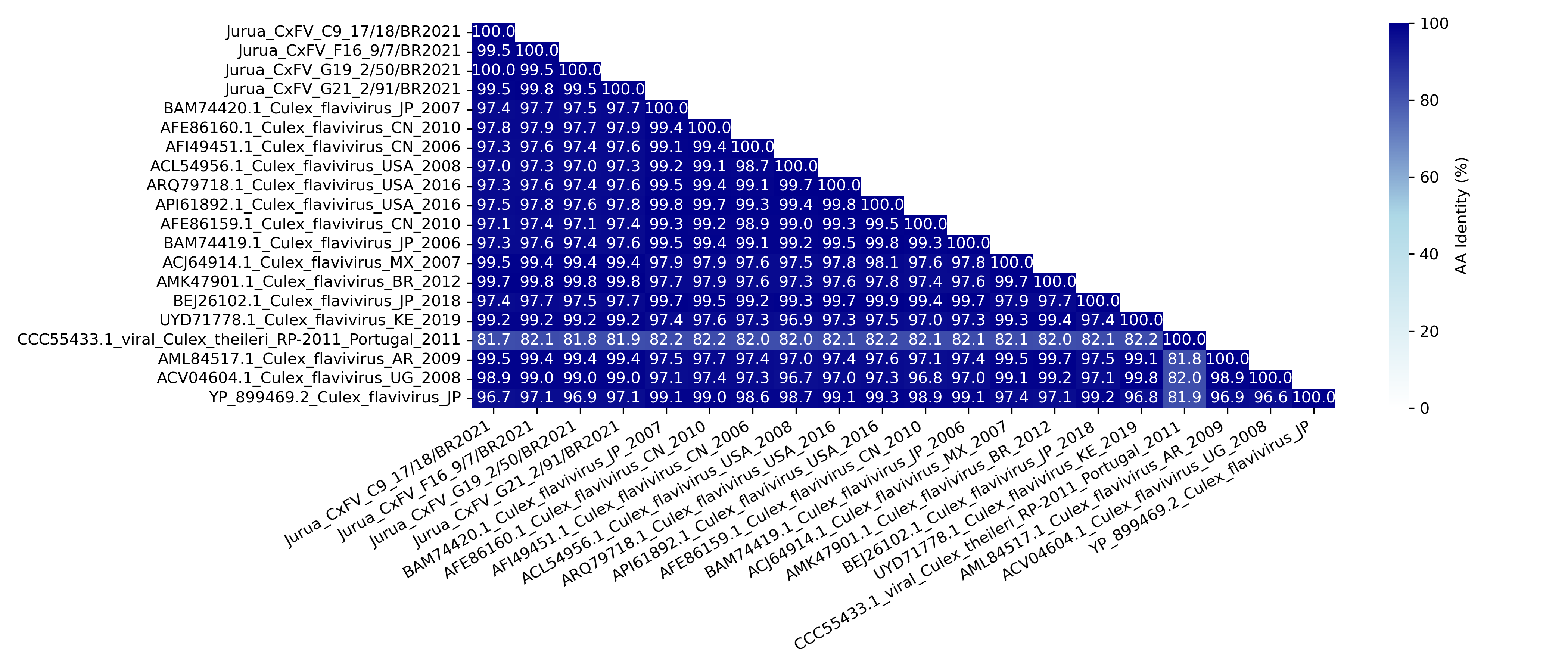

Supplement: Supplemental Information 8 — Color shading represents the degree of identity, with darker blue indicating higher similarity. [file peerj-14-20880-s008.png]

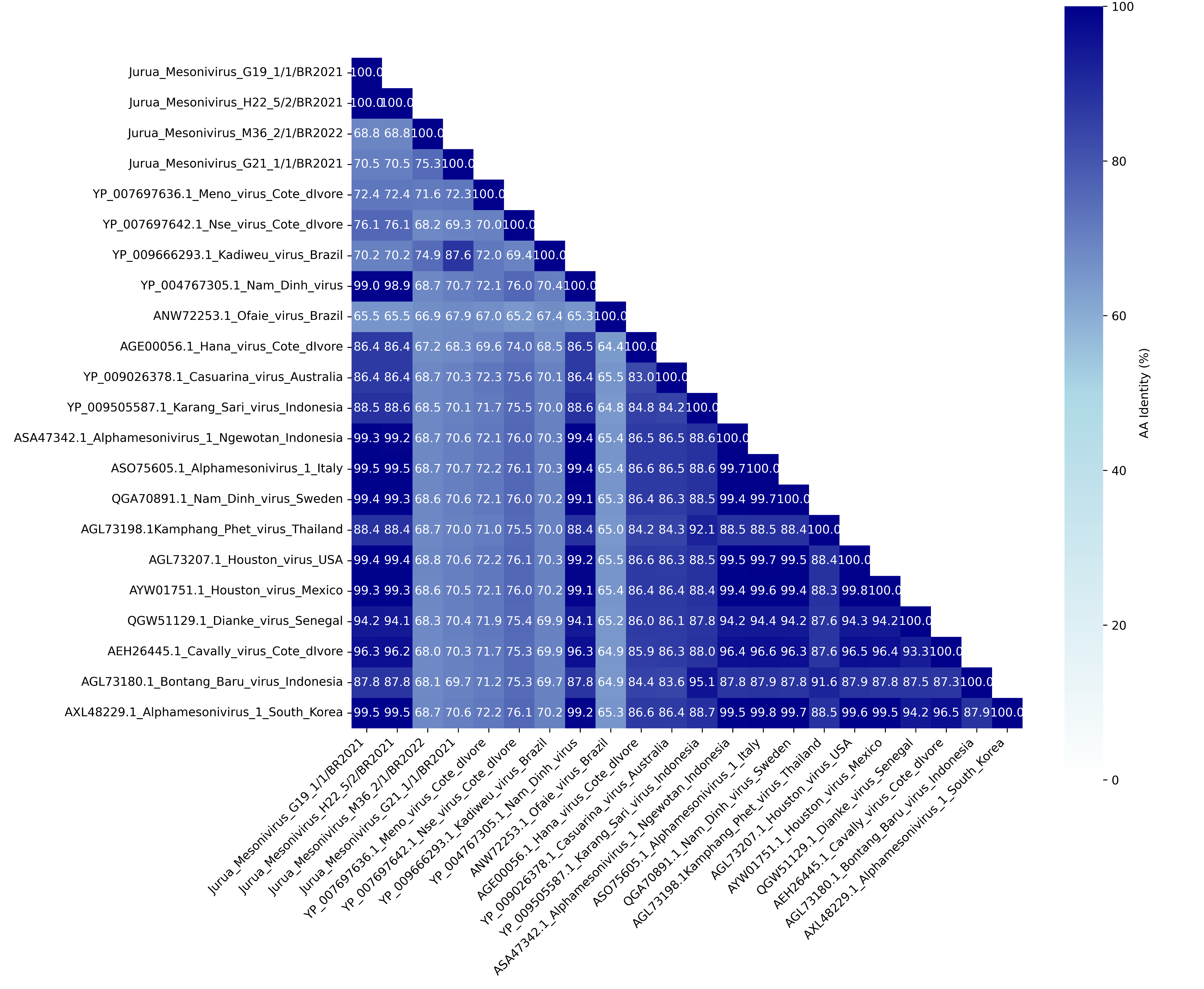

Supplement: Supplemental Information 9 — Color shading represents the degree of identity, with darker blue indicating higher similarity. [file peerj-14-20880-s009.png]

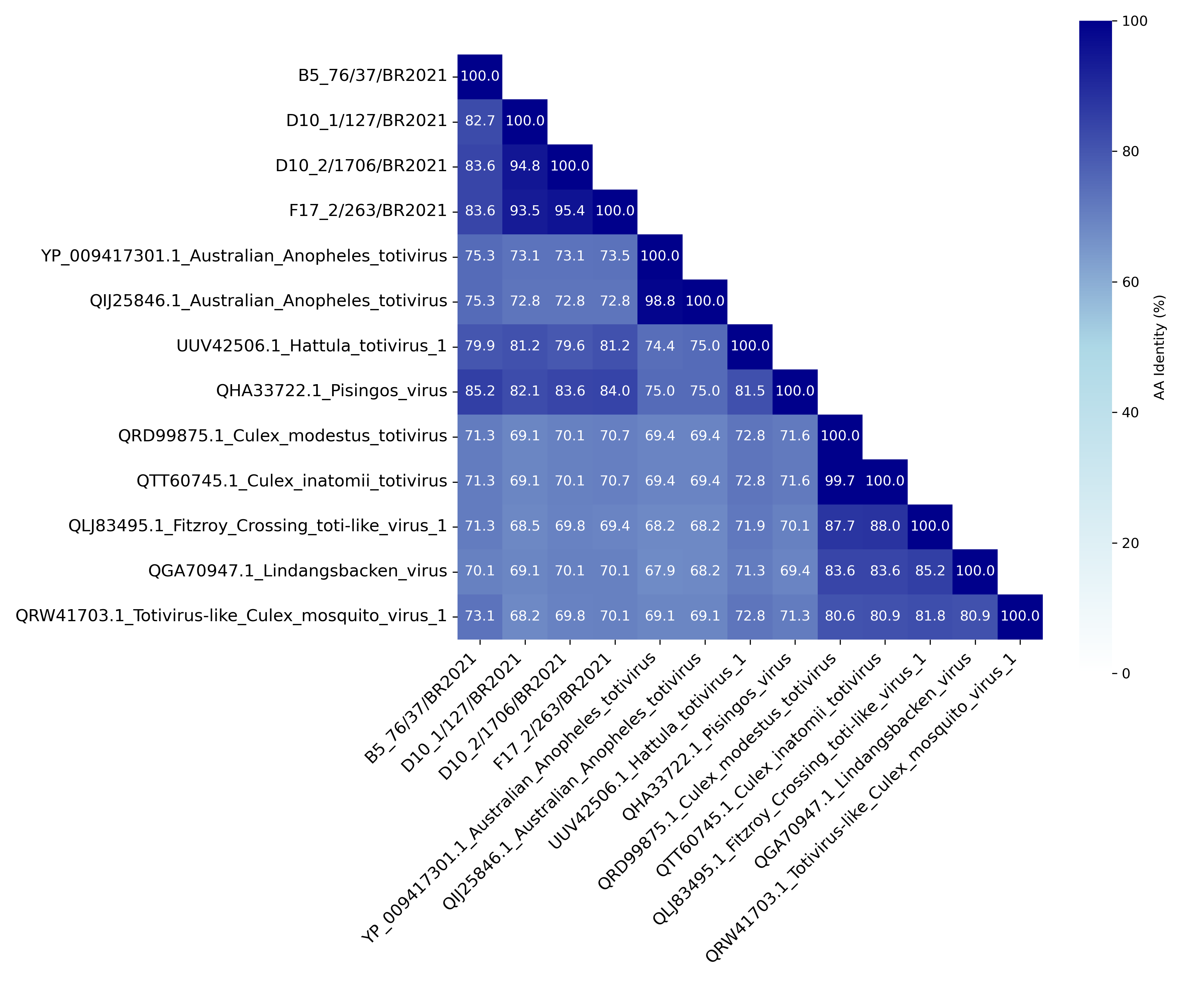

Supplement: Supplemental Information 10 — Color shading represents the degree of identity, with darker blue indicating higher similarity. [file peerj-14-20880-s010.png]
